# Supplementary material for: Shared genetic architecture between gastro-esophageal reflux disease, asthma, and allergic diseases
Source: Commun Biol. 2024 Sep 2;7:1077. doi: 10.1038/s42003-024-06795-1 (PMC11369275; doi:10.1038/s42003-024-06795-1)

Supplementary Materials

**Shared genetic origin between gastro-esophageal reflux disease,**

**asthma and allergic diseases**

Tong Gong^1^, Ralf Kuja-Halkola^1^, Arvid Harder^1^, Cecilia Lundholm^1^, Awad I. Smew^1^, Kelli Lehto^2^, Anna Andreasson^3^, Yi Lu^1^, Nicholas J. Talley^4^, Joëlle Pasman^1^, Catarina Almqvist^1,5^, Bronwyn K. Brew ^1,6^

1. Department of Medical Epidemiology and Biostatistics, Karolinska Institutet, Stockholm, Sweden.

2. Estonian Genome Centre, Institute of Genomics, University of Tartu, Tartu, Estonia

3. Stress Research Institute, Department of Psychology, Stockholm University, Stockholm, Sweden

4. School of Medicine and Public Health, University of Newcastle, Newcastle, Australia

5. Pediatric Allergy and Pulmonology Unit at Astrid Lindgren Children’s Hospital, Karolinska University Hospital, Stockholm, Sweden

6. Centre for Big Data Research in Health & School of Clinical Medicine, UNSW, Sydney, Australia

Contents

[**Supplementary Tables** 4](#_Toc175653189)

[**Supplementary Table 1. Study participants' characteristics** 4](#_Toc175653190)

[**Supplementary Table 2. Bivariate associations between asthma/allergic traits and GERD.** 6](#_Toc175653191)

[**Supplementary Table 3. Univariate and bivariate correlations for GERD and asthma/allergic traits by sex. Intraclass and phenotypic correlations are within person, CTCT are within twin pair.** 7](#_Toc175653192)

[**Supplementary Table 4. Quantitative genetic modelling - Bivariate Cholesky models. Genetic and environmental parameter estimates for Asthma and GERD using 14 197 pairs of twins.** 8](#_Toc175653193)

[**Supplementary Table 5. Quantitative genetic modelling - Bivariate Cholesky models. Genetic and environmental parameter estimates for Allergic Rhinitis and GERD using 14 197 pairs of twins.** 9](#_Toc175653194)

[**Supplementary Table 6. Quantitative genetic modelling - Bivariate Cholesky models. Genetic and environmental parameter estimates for Eczema and GERD using 14 197 pairs of twins.** 10](#_Toc175653195)

[**Supplementary Table 7. Prediction of the traits based on polygenic risk scores (PRS) in each target set.** 11](#_Toc175653196)

[**Supplementary Table 8. Associations between GERD-PRS with allergic diseases and between allergic disease-PRS with GERD among all twins.** 12](#_Toc175653197)

[**Supplementary Table 9. SNP-based heritability (h^2^_SNP_) and genetic correlation (r_g_) estimates of asthma/allergic traits and GERD. Bonferroni-corrected threshold of *p*-value = 0.01 (0.05/5 bivariate analyses).** 13](#_Toc175653198)

[**Supplementary Table 10. Results for the common factor model fit in Genomic SEM.** 14](#_Toc175653199)

[**Supplementary Table 11. Bidirectional causal relationships between asthma/allergic traits and GERD based on the two-sample Mendelian Randomization analyses.** **Bonferroni-corrected threshold of *p* = 0.008 (0.05/6 traits).** 15](#_Toc175653200)

[**Supplementary Tables 12-17 are presented via Figshare.** 16](#_Toc175653201)

[**Supplementary figures** 17](#_Toc175653202)

[**Supplementary Figure 1. Genetic correlations between all traits, with the correlation in the lower triangle and the corresponding standard error in the top triangle. Results from LD score regression.** 17](#_Toc175653203)

[**Supplementary Figure 2. Prediction of the traits based on polygenic risk scores in deciles among each target set.** 18](#_Toc175653204)

[**Supplementary Figure 3. Scatter plot and leave-one-out analysis for the association of asthma (instrumental variable, IV) with GERD (outcome)** 21](#_Toc175653205)

[**Supplementary Figure 4. Scatter plot and leave-one-out analysis for the association of GERD (IV) with asthma (outcome).** 24](#_Toc175653206)

[**Supplementary Figure 5. Scatter plot and leave-one-out analysis for the association of eczema (IV) with GERD (outcome).** 27](#_Toc175653207)

[**Supplementary Figure 6. Scatter plot and leave-one-out analysis for the association of allergic rhinitis (IV) with GERD (outcome).** 30](#_Toc175653208)

[**Supplementary Figure 7. Scatter plot and leave-one-out analysis for the association of GERD (IV) with allergic rhinitis (outcome).** 33](#_Toc175653209)

[**Supplementary Figure 8. Scatter plot and leave-one-out analysis for the association of GERD (IV) with eczema (outcome).** 36](#_Toc175653210)

[**Supplementary Figure 9. Manhattan plot (gene-based test) for GERD.** 39](#_Toc175653211)

[**Supplementary Figure 10. Manhattan plot (gene-based test) for asthma.** 40](#_Toc175653212)

[**Supplementary Figure 11. Manhattan plot (gene-based test) for allergic rhinitis.** 41](#_Toc175653213)

[**Supplementary Figure 12. Manhattan plot (gene-based test) for eczema** 42](#_Toc175653214)

[**Supplementary Figure 13. Regional association plot for asthma (up) and GERD (down) close to RAB5B and ERBB3 genes.** 43](#_Toc175653215)

[**Supplementary Figure 14. Regional association plot for asthma (up) and GERD (down) close to RBM6 gene.** 44](#_Toc175653216)

[**Supplementary Figure 15. Regional association plot for asthma (up) and GERD (down) close to SDK1 gene.** 45](#_Toc175653217)

[**Supplementary Figure 16. Regional association plot for asthma (up) and GERD (down) close to HLA-B gene.** 46](#_Toc175653218)

[**Supplementary Figure 17. Regional association plot for asthma (up) and GERD (down) close to RERG gene.** 47](#_Toc175653219)

[**Supplementary Figure 18. MAGMA Tissue Expression Analysis of GERD and plotting differentially expressed genes in relevant tissues (GTEx v8 30 general tissue types).** 48](#_Toc175653220)

[**Supplementary Figure 19. MAGMA Tissue Expression Analysis of asthma and plotting differentially expressed genes in relevant tissues (GTEx v8 30 general tissue types)** 51](#_Toc175653221)

[**Supplementary Figure 20. MAGMA Tissue Expression Analysis of allergic rhinitis and plotting differentially expressed genes in relevant tissues (GTEx v8 30 general tissue types)** 52](#_Toc175653222)

[**Supplementary Figure 21. MAGMA Tissue Expression Analysis of eczema and plotting differentially expressed genes in relevant tissues (GTEx v8 30 general tissue types)** 53](#_Toc175653223)

# **Supplementary Tables**

## **Supplementary Table 1. Study participants' characteristics**

|  | **STAGE**  **(n=13742)** | **TwinGene (n=11875)** | **SALTY**  **(n=2777)** | **All**  **(n= 28394)** |
| --- | --- | --- | --- | --- |
| **Born 1911-1938** | 0 | 3746(31.55) | 0 | 3746 (13.19) |
| **Born 1939-1948** | 0 | 5380(45.31) | 736(26.50) | 6116 (21.54) |
| **Born 1949-1958** | 0 | 2749(23.15) | 2041(73.50) | 4790 (16.87) |
| **Born 1959-1968** | 5454(39.69) | 0 | 0 | 5454 (19.21) |
| **Born 1969-1978** | 4966(36.14) | 0 | 0 | 4966 (17.49) |
| **Born 1979-1985** | 3322(24.17) | 0 | 0 | 3322 (11.70) |
| **Zygosity** |  |  |  |  |
| MZ twins | 5856(42.61) | 3159(26.60) | 1011(36.41) | 10026(35.31) |
| Same-sex DZ twins | 3996(29.08) | 4602(38.75) | 930(33.49) | 9528(33.56) |
| Opposite-sex DZ twins | 3890(28.31) | 4114(34.64) | 836(30.10) | 8840(31.13) |
| **Sex** |  |  |  |  |
| Male | 5669(41.25) | 5390(45.39) | 1225(44.11) | 12284(43.26) |
| Female | 8073(58.75) | 6485(54.61) | 1552(55.89) | 16110(56.74) |
| **Smoking** |  |  |  |  |
| Yes | 3476(25.29) | 1972(16.61) | 548(19.73) | 5996(21.12) |
| **Education** |  |  |  |  |
| Compulsory school | 658(4.79) | 5784(48.71) | 875(31.51) | 7317(25.77) |
| High school | 5830(42.42) | 2102(17.70) | 756(27.22) | 8688(30.60) |
| University or higher education | 6117(44.51) | 3347(28.19) | 957(34.46) | 10421(36.70) |
| **Mean BMI** (SD) | 23.91 (6.34) | 24.94(3.38) | 24.68(3.47) | 24.42(5.06) |
| **GERD** |  |  |  |  |
| Yes | 1813(13.19) | 1320(11.12) | 287(10.33) | 3420(12.04) |
| **Asthma** |  |  |  |  |
| Yes | 1416(10.30) | 627(5.28) | 214(7.71) | 2257(7.95) |
| **Allergic rhinitis** |  |  |  |  |
| Yes | 2226(16.20) | 733(6.17) | 267(9.61) | 3226(11.36) |
| **Eczema** |  |  |  |  |
| Yes | 822(5.98) | 901(7.59) | 261(9.40) | 1984(6.99) |

## **Supplementary Table 2. Bivariate associations between asthma/allergic traits and GERD.**

| **Exposure** | **Outcome** | **OR (95% CI)** | |
| --- | --- | --- | --- |
|  |  | **Unadjusted** | **Adjusted*** |
| **Asthma** | GERD | 1.64 (1.47, 1.84) | 1.61 (1.43, 1.80) |
| No asthma |  | ref | Ref |
|  |  |  |  |
| **Allergic Rhinitis** | GERD | 1.15 (1.03, 1.28) | 1.14 (1.02, 1.28) |
| No Allergic Rhinitis |  | ref | ref |
|  |  |  |  |
| **Eczema** | GERD | 1.19 (1.05, 1.36) | 1.14 (1.00, 1.30) |
| No eczema |  | ref | ref |
|  |  |  |  |
| **GERD** | Asthma | 1.61 (1.43, 1.80) | 1.58 (1.41, 1.77) |
| No GERD |  | ref | Ref |
|  |  |  |  |
| **GERD** | Allergic Rhinits | 1.12 (1.01, 1.25) | 1.13 (1.01, 1.27) |
| No GERD |  | ref | ref |
|  |  |  |  |
| **GERD** | Eczema | 1.19 (1.05, 1.36) | 1.14 (1.00, 1.31) |
| No GERD |  | ref | ref |

## **Supplementary Table 3. Univariate and bivariate correlations for GERD and asthma/allergic traits by sex. Intraclass and phenotypic correlations are within person, CTCT are within twin pair.**

|  | **Univariate**  **Intraclass correlations (ICC)** | | | | **Bivariate phenotypic and cross-twin cross trait (CTCT) correlations** | | | | | |
| --- | --- | --- | --- | --- | --- | --- | --- | --- | --- | --- |
|  |  |  |  |  | **Asthma & GERD** | | **Allergic Rhinitis & GERD** | | **Eczema & GERD** | |
| **Type of relatives** | **GERD** | **Asthma** | **Allergic Rhinitis** | **Eczema** | **Phenotypic correlation** | **CTCT correlation** | **Phenotypic correlation** | **CTCT correlation** | **Phenotypic correlation** | **CTCT correlation** |
| **MZ twins** |  |  |  |  |  |  |  |  |  |  |
| Females | 0.29 | 0.64 | 0.59 | 0.41 | 0.10 | 0.10 | 0.02 | 0.01 | 0.00 | 0.01 |
|  | (0.24, 0.35) | (0.60, 0.68) | (0.55, 0.63) | (0.35, 0.47) | (0.04, 0.16) | (0.04, 0.16) | (-.04, 0.08) | (-0.06, 0.07) | (-0.07, 0.06) | (-0.06, 0.08) |
| Males | 0.35 | 0.64 | 0.66 | 0.46 | 0.10 | -0.07 | 0.02 | 0.04 | -0.04 | 0.08 |
|  | (0.27, 0.43) | (0.58, 0.70) | (0.61, 0.71) | (0.37, 0.55) | (0.00, 0.20) | (-0.18, 0.04) | (-.07, 0.11) | (-0.05, 0.13) | (-0.16, 0.08) | (-0.03, 0.19) |
| **DZ twins** |  |  |  |  |  |  |  |  |  |  |
| Females | 0.20 | 0.30 | 0.30 | 0.18 | 0.11 | 0.04 | 0.10 | 0.11 | 0.00 | 0.00 |
|  | (0.14, 0.26) | (0.23, 0.37) | (0.24, 0.37) | (0.10, 0.26) | (0.04, 0.18) | (-0.03, 0.12) | (0.03, 0.16) | (0.04, 0.17) | (-.07, 0.08) | (-0.08, 0.07) |
| Males | 0.14 | 0.39 | 0.40 | 0.09 | 0.18 | 0.05 | -0.02 | -0.04 | 0.01 | -0.04 |
|  | (0.06, 0.23) | (0.30, 0.48) | (0.30, 0.47) | (-0.04, 0.21) | (0.08, 0.28) | (-0.05, 0.16) | (-0.12, 0.07) | (-0.13, 0.06) | (-.10, 0.12) | (-0.16, 0.07) |
| Opposite sex | 0.01 | 0.25 | 0.25 | 0.13 | 0.18 | 0.02 | 0.10 | -0.01 | 0.07 | 0.04 |
|  | (-0.04, 0.07) | (0.19, 0.31) | (0.20, 0.30) | (0.06, 0.20) | (0.12, 0.24) | (-0.04, 0.08) | (0.04, 0.15) | (-0.06, 0.05) | (0.01, 0.13) | (-.02, 0.11) |

## **Supplementary Table 4. Quantitative genetic modelling - Bivariate Cholesky models. Genetic and environmental parameter estimates for Asthma and GERD using 14 197 pairs of twins.**

|  | Models | | |
| --- | --- | --- | --- |
| Parameters | ACE | ADE | AE |
| *Asthma, % (95% CI)* |  |  |  |
| Asthma due to A | 0.61 (0.57, 0.66) | 0.49 (0.22, 0.76) | 0.61 (0.57, 0.66) |
| Asthma due to D | Na | 0.14 (-0.15, 0.42) | Na |
| Asthma due to H | Na | 0.63 (0.57, 0.68) | Na |
| Asthma due to C | 0.00 (0.00, 0.00) | Na | Na |
| Asthma due to E | 0.39 (0.34, 0.43) | 0.37 (0.32, 0.43) | 0.39 (0.34, 0.43) |
| *GERD, % (95% CI)* |  |  |  |
| GERD trait due to A | 0.29 (0.24, 0.34) | 0.11 (0.05, 0.17) | 0.29 (0.24, 0.34) |
| GERD trait due to D | Na | 0.20 (0.14, 0.27) | Na |
| GERD trait due to H | Na | 0.31 (0.25, 0.37) | Na |
| GERD trait due to C | 0.00 (0.00, 0.00) | Na | Na |
| GERD trait due to E | 0.71 (0.66, 0.76) | 0.69 (0.63, 0.75) | 0.71 (0.66, 0.76) |
| *Bivariate explained covariance, % (95% CI)* |  |  |  |
| Covariance due to A | 0.56 (0.26, 0.87) | 0.39 (0.12, 0.66) | 0.56 (0.26, 0.87) |
| Covariance due to D | Na | 0.20 (-0.10, 0.51) | Na |
| Covariance due to H | Na | 0.59 (0.23, 0.96) | Na |
| Covariance due to C | 0.00 (0.00, 0.00) | Na | Na |
| Covariance due to E | 0.44 (0.13, 0.74) | 0.41 (0.04, 0.77) | 0.44 (0.13, 0.74) |
| *Bivariate correlations, % (95% CI)* |  |  |  |
| rA | 0.18 (0.08, 0.28) | 0.23 (0.07, 0.38) | 0.18 (0.08, 0.28) |
| rD | Na | 0.16 (-0.13, 0.46) | Na |
| rH | Na | 0.28 (0.11, 0.44) | Na |
| rC | 0.60 (Na, Na) | Na | Na |
| rE | 0.11 (0.03, 0.20) | 0.11 (0.01, 0.21) | 0.11 (0.03, 0.20) |
| *Model fitting* |  |  |  |
| AIC | 35659.76 | 35656.04 | 35653.76 |
| p-values from likelihood ratio test |  |  |  |
| ACE VS AE | 1 | | |
| ADE VS AE | 0.293 | | |

Notes:

1. After running the saturated model and several assumption models, we used the base model with equal threshold to build the ACE, ADE, AE models. We used a weighted least squares approach to model the point estimate and the 95% confidence intervals (CI) based on standard errors (Wald CI). Therefore, the 95% CIs might fall above 1 or below -1 for correlations, and below 0 for variance components.

2. All models were adjusted for sex and birth year (continuous, standardized).

Abbreviations: A, additive genetic component; D, non-additive/ dominant genetic component; H, broad-sense heritability component, which is A+D; C, shared environmental component; E, non-shared environmental component (including measurement errors).

## **Supplementary Table 5. Quantitative genetic modelling - Bivariate Cholesky models. Genetic and environmental parameter estimates for Allergic Rhinitis and GERD using 14 197 pairs of twins.**

|  | Models | | |
| --- | --- | --- | --- |
| Parameters | ACE | ADE | AE |
| *Allergic rhinitis, % (95% CI)* |  |  |  |
| Allergic rhinitis due to A | 0.58 (0.53,0.62) | 0.40 (na, na) | 0.58 (0.53,0.62) |
| Allergic rhinitis due to D | na | 0.20 (na, na) | na |
| Allergic rhinitis due to H | na | 0.59 (0.55, 0.64) | na |
| Allergic rhinitis due to C | 0.00 (0.00, 0.00) | na | na |
| Allergic rhinitis due to E | 0.42 (0.38, 0.47) | 0.41 (0.36, 0.45) | 0.42 (0.38, 0.47) |
| *GERD, % (95% CI)* |  |  |  |
| GERD trait due to A | 0.29 (0.23, 0.34) | 0.11 (0.01, 0.21) | 0.29 (0.23, 0.34) |
| GERD trait due to D | Na | 0.20 (0.08, 0.33) | na |
| GERD trait due to H | Na | 0.31 (0.25, 0.37) | na |
| GERD trait due to C | 0.00 (0.00, 0.00) | Na | na |
| GERD trait due to E | 0.71 (0.66, 0.77) | 0.69 (0.63, 0.75) | 0.71 (0.66, 0.77) |
| *Bivariate explained covariance, % (95% CI)* |  |  |  |
| Covariance due to A | 0.61 (0.03, 1.19) | 1.02 (0.08, 1.96) | 0.61 (0.03, 1.19) |
| Covariance due to D | Na | -0.46 (-1.43, 0.51) | na |
| Covariance due to H | Na | 0.56 (0.03, 1.09) | na |
| Covariance due to C | 0.00 (0.00, 0.00) | na | na |
| Covariance due to E | 0.39 (-0.19, 0.97) | 0.44 (-0.09, 0.97) | 0.39 (-0.19, 0.97) |
| *Bivariate correlations, % (95% CI)* |  |  |  |
| rA | 0.10 (0.00, 0.19) | 0.31 (0.03, 0.59) | 0.10 (0.00, 0.19) |
| rD | na | -0.15 (-0.43, 0.14) | na |
| rH | na | 0.17 (-0.01, 0.35) | na |
| rC | -1.00 (-51.06, 49.06) | na | na |
| rE | 0.05 (-0.03, 0.12) | 0.05 (-0.02, 0.12) | 0.05 (-0.03, 0.12) |
| *Model fitting* |  |  |  |
| AIC | 39590.86 | 39585.12 | 39584.86 |
| p-values from likelihood ratio test |  |  |  |
| ACE VS AE | 1 | | |
| ADE VS AE | 0.125 | | |

Notes:

1. After running the saturated model and several assumption models, we used the base model with equal threshold to build the ACE, ADE, AE models. We used a weighted least squares approach to model the point estimate and the 95% confidence intervals (CI) based on standard errors (Wald CI). Therefore, the 95% CIs might fall above 1 or below -1 for correlations, and below 0 for variance components.

2. All models were adjusted for sex and birth year (continuous, standardized).

Abbreviations: A, additive genetic component; D, non-additive/ dominant genetic component; H, broad-sense heritability component, which is A+D; C, shared environmental component; E, non-shared environmental component (including measurement errors).

## **Supplementary Table 6. Quantitative genetic modelling - Bivariate Cholesky models. Genetic and environmental parameter estimates for Eczema and GERD using 14 197 pairs of twins.**

|  | Models | | |
| --- | --- | --- | --- |
| Parameters | ACE | ADE | AE |
| *Eczema, % (95% CI)* |  |  |  |
| Eczema due to A | 0.40 (0.33, 0.46) | 0.16 (-0.12, 0.44) | 0.40 (0.33, 0.46) |
| Eczema due to D | na | 0.27 (-0.04, 0.58) | na |
| Eczema due to H | na | 0.43 (0.36, 0.50) | na |
| Eczema due to C | 0.00 (0.00, 0.00) | na | na |
| Eczema due to E | 0.60 (0.54, 0.67) | 0.57 (0.50, 0.64) | 0.60 (0.54, 0.67) |
| *GERD, % (95% CI)* |  |  |  |
| GERD trait due to A | 0.29 (0.23, 0.34) | 0.11 (-0.11, 0.33) | 0.29 (0.23, 0.34) |
| GERD trait due to D | na | 0.20 (-0.05, 0.45) | na |
| GERD trait due to H | na | 0.31 (0.25, 0.38) | na |
| GERD trait due to C | 0.00 (0.00, 0.00) | na | na |
| GERD trait due to E | 0.71 (0.66, 0.77) | 0.69 (0.62, 0.75) | 0.71 (0.66, 0.77) |
| *Bivariate explained covariance, % (95% CI)* |  |  |  |
| Covariance due to A | 1.77 (-1.80, 5.34) | 0.89 (-9.53, 10.91) | 1.77 (-1.80, 5.34) |
| Covariance due to D | na | 0.83 (-10.35, 12.92) | na |
| Covariance due to H | na | 1.98 (-2.21, 6.17) | na |
| Covariance due to C | 0.00 (0.00, 0.00) | na | na |
| Covariance due to E | -0.77 (-4.34, 2.80) | -0.98 (-5.17, 3.21) | -0.77 (-4.34, 2.80) |
| *Bivariate correlations, % (95% CI)* |  |  |  |
| rA | 0.10 (-0.04, 0.25) | 0.10 (-1.41, 1.61) | 0.10 (-0.04, 0.25) |
| rD | na | 0.11 (-0.86, 1.08) | Na |
| rH | na | 0.15 (-0.11, 0.40) | Na |
| rC | -1.00 (-2260.78, 2258.78) | Na | Na |
| rE | -0.02 (-0.10, 0.06) | -0.03 (-0.13, 0.07) | -0.02 (-0.10, 0.06) |
| *Model fitting* |  |  |  |
| AIC | 34865.01 | 34859.26 | 34859.01 |
| p-values from likelihood ratio test |  |  |  |
| ACE VS AE | 1 | | |
| ADE VS AE | 0.124 | | |

Notes:

1. After running the saturated model and several assumption models, we used the base model with equal threshold to build the ACE, ADE, AE models. We used a weighted least squares approach to model the point estimate and the 95% confidence intervals (CI) based on standard errors (Wald CI). Therefore, the 95% CIs might fall above 1 or below -1 for correlations, and below 0 for variance components.

2. All models were adjusted for sex and birth year (continuous, standardized).

Abbreviations: A, additive genetic component; D, non-additive/ dominant genetic component; H, broad-sense heritability component, which is A+D; C, shared environmental component; E, non-shared environmental component (including measurement errors).

## **Supplementary Table 7. Prediction of the traits based on polygenic risk scores (PRS) in each target set.**

| Phenotype | PRS (sumstat source) | STAGE | | | TwinGene | | | SALTY | | |
| --- | --- | --- | --- | --- | --- | --- | --- | --- | --- | --- |
|  |  | n_Case_: n_control_ | Nagelkerke R^2^ | AUC | n_Case_: n_control_ | Nagelkerke R^2^ | AUC | n_Case_: n_control_ | Nagelkerke R^2^ | AUC |
| Asthma | Asthma (Zhou et al.) | 892/7903 | 0.056 | 0.6522 | 603/10226 | 0.039 | 0.6417 | 393/4898 | 0.059 | 0.6652 |
| Asthma | Childhood-onset asthma (COA, Ferriera et al) | 892/7903 | 0.051 | 0.6448 | 603/10226 | 0.030 | 0.6228 | 393/4898 | 0.028 | 0.6066 |
| Asthma | Adulthood-onset asthma (AOA, Ferriera et al) | 892/7903 | 0.031 | 0.6099 | 603/10226 | 0.029 | 0.6262 | 393/4898 | 0.029 | 0.6151 |
| Allergic rhinitis | Allergic rhinitis (Waage et al) | 1460/7393 | 0.002 | 0.5212 | 622/10069 | 0.004 | 0.5410 | 480/4737 | 0.001 | 0.5245 |
| Eczema | Eczema (Sliz et al) | 534/8274 | 0.026 | 0.6135 | 652/7734 | 0.009 | 0.5628 | 324/3490 | 0.010 | 0.5617 |
| GERD | GERD (An et al) | 1232/7836 | 0.011 | 0.5655 | 1220/9574 | 0.012 | 0.5671 | 616/5112 | 0.011 | 0.5655 |

Note**:** AUC-area under the curve.

## **Supplementary Table 8. Associations between GERD-PRS with allergic diseases and between allergic disease-PRS with GERD among all twins.**

| Phenotype | PRS | Number of cases | Sample size | OR (95% CI) | |
| --- | --- | --- | --- | --- | --- |
|  |  |  |  | Model 1 | Model 2 |
| Asthma | GERD | 1888 | 26895 | 1.14 (1.09, 1.20) | 1.14 (1.08, 1.20) |
| Allergic rhinitis | GERD | 2562 | 26895 | 1.08 (1.03, 1.12) | 1.08 (1.03, 1.13) |
| Eczema | GERD | 1510 | 26895 | 1.01 (0.96, 1.07) | 1.01 (0.96, 1.07) |
|  |  |  |  |  |  |
| GERD | Asthma | 3068 | 26895 | 1.09 (1.05, 1.14) | 1.09 (1.05, 1.14) |
| GERD | COA | 3068 | 26895 | 0.99 (0.95, 1.03) | 0.99 (0.95, 1.03) |
| GERD | AOA | 3068 | 26895 | 1.04 (1.00, 1.08) | 1.04 (1.00, 1.08) |
| GERD | Allergic rhinitis | 3068 | 26895 | 0.99 (0.95, 1.03) | 0.99 (0.95, 1.03) |
| GERD | Eczema | 3068 | 26895 | 1.02 (0.98, 1.06) | 1.02 (0.98, 1.06) |

Model 1 with no adjustment.

Model 2 adjusted for birth year, sex, cohort, top 5 principal components, and cohort*top 5 principal components.

## **Supplementary Table 9. SNP-based heritability (h^2^_SNP_) and genetic correlation (r_g_) estimates of asthma/allergic traits and GERD. Bonferroni-corrected threshold of *p*-value = 0.01 (0.05/5 bivariate analyses).**

|  | **N**  **(sample prevalence %,**  **population prevalence %)** | **Nr. of SNPs remained in the analysis** | **h^2^_SNP_ (CI) ^1^** | **Intercept (SE) ^2^** | **Ratio (SE) ^3^** |
| --- | --- | --- | --- | --- | --- |
| ***Univariate analyses*** |  |  |  |  |  |
| Asthma | 1800785 (8.5%, 8%) | 958562 | 0.079 (0.071, 0.087) | 1.0886 (0.0122) | 0.1162 (0.016) |
| Adulthood onset asthma | 327253 (8.1%, 8%) | 1168364 | 0.126 (0.107, 0.145) | 1.0366 (0.0087) | 0.1284 (0.0307) |
| Childhood onset asthma | 314633 (4.4%, 5%) | 1168396 | 0.298 (0.194, 0.402) | 1.0644 (0.0114) | 0.1446 (0.0256) |
| Allergic rhinitis | 38838 (27.2%, 25%) | 1174226 | 0.119 (0.072, 0.166) | 0.9912 (0.007) | < 0 |
| Eczema | 796661 (2.8%, 3%) | 1168494 | 0.078 (0.048, 0.109) | 1.0423 (0.0088) | 0.3549 (0.0736) |
| GERD | 332601 (21.5%, 20%) | 1180258 | 0.131 (0.121, 0.141) | 1.0401 (0.0082) | 0.0843 (0.0173) |
|  |  |  |  |  |  |
| ***Bivariate analyses*** | **h^2^ _SNP (trait 1):_ h^2^ _SNP (trait 2)_** | **r_g_ (CI)** | ***p*-value** | **Intercept _(trait 1):_ Intercept _(trait 2)_** | **Ratio _(trait 1):_ Ratio _(trait 2)_** |
| Asthma-GERD | 0.0626: 0.1329 | 0.475 (0.424, 0.525) | 7.8256e-75 | 1.0672:1.0404 | 0.088: 0.0847 |
| Adulthood onset asthma-GERD | 0.1293:0.1311 | 0.330 (0.269, 0.392) | 8.3652e-26 | 1.0314:1.0409 | 0.1101: 0.0857 |
| Childhood onset asthma-GERD | 0.3303: 0.1311 | 0.075 (0.029, 0.122) | 0.0015 | 1.0325:1.0409 | 0.0729:0.0857 |
| Allergic rhinitis-GERD | 0.1165:0.1317 | 0.145 (0.015, 0.274) | 0.0286 | 0.9921:1.0385 | <0:0.0804 |
| Eczema-GERD | 0.082:0.1314 | 0.200 (0.105, 0.295) | 3.4872e-05 | 1.0385:1.0395 | 0.3239: 0.0835 |

## **Supplementary Table 10. Results for the common factor model fit in Genomic SEM.**

| **Trait 1** | **Operator** | **Trait 2** | $\beta$ | **SE** | ***p-*value** |
| --- | --- | --- | --- | --- | --- |
| Common factor | =~ | Adult onset asthma | 1 | * | * |
| Common factor | =~ | Childhood onset asthma | 0.765 | 0.063 | 1.35E-33 |
| Common factor | =~ | Allergic rhinitis | 0.838 | 0.0940 | 5.08E-19 |
| Common factor | =~ | Eczema | 0.852 | 0.099 | 6.11E-18 |
| Common factor | =~ | GERD | 0.237 | 0.031 | 2.13E-14 |
| Adult onset asthma | ~~ | Adult onset asthma | 0.095 | 0.091 | 0.297 |
| Childhood onset asthma | ~~ | Childhood onset asthma | 0.470 | 0.070 | 1.77E-11 |
| Allergic rhinitis | ~~ | Allergic rhinitis | 0.365 | 0.201 | 0.069 |
| Eczema | ~~ | Eczema | 0.342 | 0.168 | 0.042 |
| Common factor | ~~ | Common factor | 0.905 | 0.110 | 1.49E-16 |

* First indicator was fixed to 1; no SE and p-value are available

=~ factor is measured by; x ~~ y covariance; x ~~ x residual variance

$\beta$ = standardized genotype; SE = standard error

## **Supplementary Table 11. Bidirectional causal relationships between asthma/allergic traits and GERD based on the two-sample Mendelian Randomization analyses.** **Bonferroni-corrected threshold of *p* = 0.008 (0.05/6 traits).**

| **Exposure-Outcome** | **Number of SNPs as instrument variables** | **Mean F-statistics** | **MR-Egger intercept** | ***p*-value for the MR-Egger intercept test** | ***p*-value for MR-Egger Q heterogeneity test** | **OR (95% CI) by IVW approach** | ***p*-value by IVW approach** | ***p*-value for IVW Q heterogeneity test** |
| --- | --- | --- | --- | --- | --- | --- | --- | --- |
| Asthma-GERD | 107 | 74.21 | 0.005 | 0.06 | 0 | 1.09 (1.05, 1.14) | <0.001 | 0 |
| Allergic rhinitis-GERD | 3 | 38.92 | -0.014 | 0.58 | 0.55 | 0.99 (0.93, 1.05) | 0.71 | 0.62 |
| Eczema-GERD | 19 | 54.44 | 0.010 | 0.16 | 0.03 | 0.92 (0.70, 1.15) | 0.48 | 0.02 |
| GERD-Asthma | 18 | 33.48 | -0.010 | 0.78 | 0 | 1.27 (1.12, 1.43) | 0.002 | 0 |
| GERD-Allergic rhinitis | 23 | 33.96 | 0.003 | 0.92 | 0.17 | 0.81 (0.58, 1.05) | 0.08 | 0.21 |
| GERD-Eczema | 18 | 33.56 | 0.006 | 0.84 | 0.0003 | 0.97 (0.75, 1.18) | 0.75 | 0.0005 |

## **Supplementary Tables 12-17 are presented via Figshare.**

Gong, Tong; Brew, Bronwyn (2023): Supplementary tables S12-S17. figshare. Dataset. <https://figshare.com/articles/online_resource/TableS12_S17_xlsxGong_Supplementary_Tables_S12-S17/26860399?file=48842842>

Excel sheet name: Table S12.

• Description: Asthma MAGMA gene-based association- source data for Figure S19.

Excel sheet name: Table S13.

• Description: Allergic rhinitis MAGMA gene-based association- source data for Figure S20.

Excel sheet name: Table S14.

• Description: Eczema MAGMA gene-based association- source data for Figure S21.

Excel sheet name: Table S15.

• Description: GERD MAGMA gene-based association- source data for Figure S18.

Excel sheet name: Table S16.

• Description: A compilation of reported associations at the loci 12q13.2 from GWAS Catalog.

Excel sheet name: Table S17.

• Description: Results of gene sets enrichment analysis for each allergic disease (i.e. asthma, allergic rhinitis, and eczema) and GERD using MAGMA.

# **Supplementary figures**

## **Supplementary Figure 1. Genetic correlations between all traits, with the correlation in the lower triangle and the corresponding standard error in the top triangle. Results from LD score regression.**


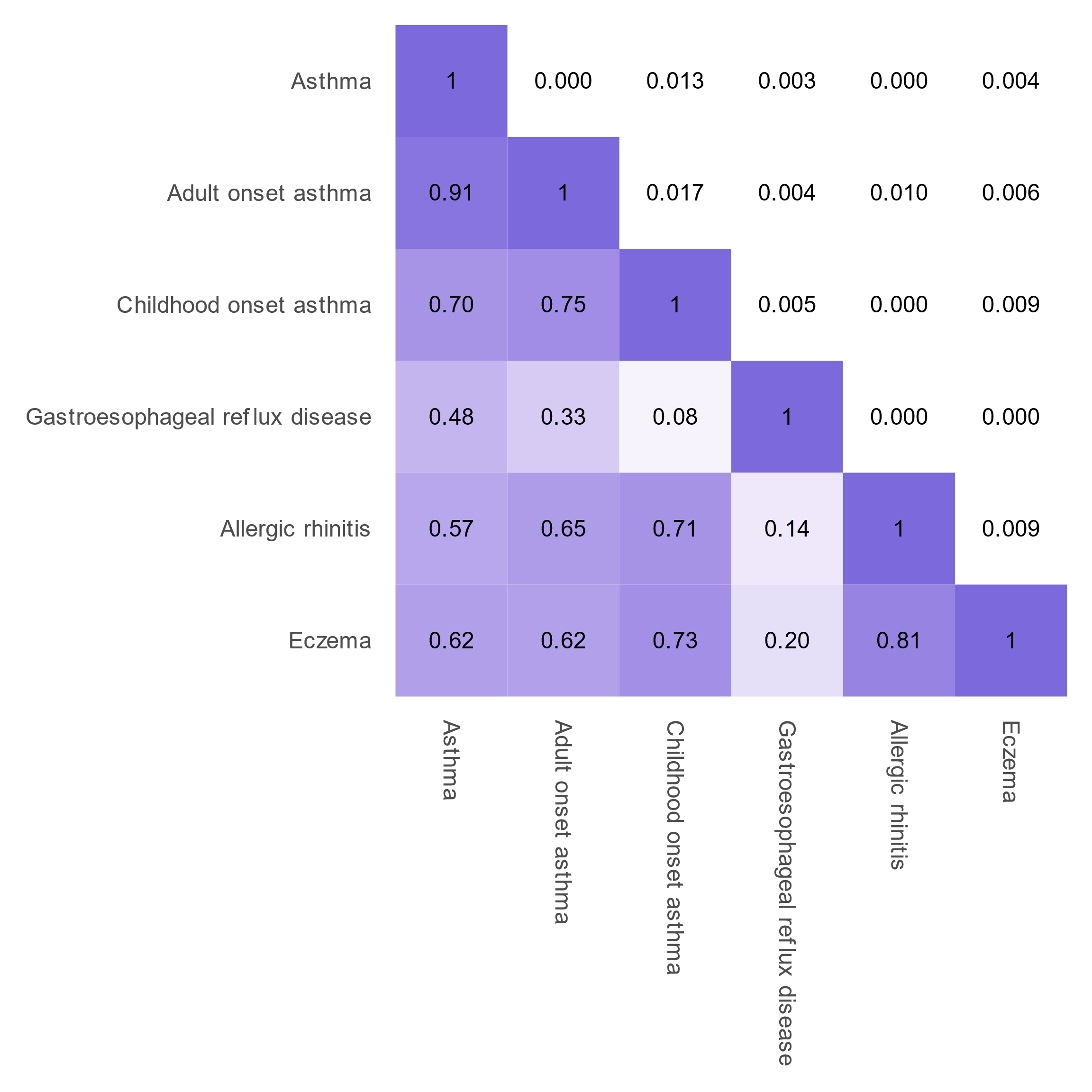


## **Supplementary Figure 2. Prediction of the traits based on polygenic risk scores in deciles among each target set.**

Figure Legend: The associations presented in odds ratios (OR) are statistically significant when the horizontal line of the confidence interval (error bars) does not cross the vertical red line at the value 1. Comparing the bottom decile for the polygenic score, the top decile of polygenic score for asthma (including childhood-onset asthma and adulthood-onset asthma), allergic rhinitis, eczema, and GERD is associated with odds ratios of 4-7, 0.6-0.9, 2-4, and 2 for self-reported traits of doctor-diagnosed asthma, allergic rhinitis, eczema, and GERD symptoms.


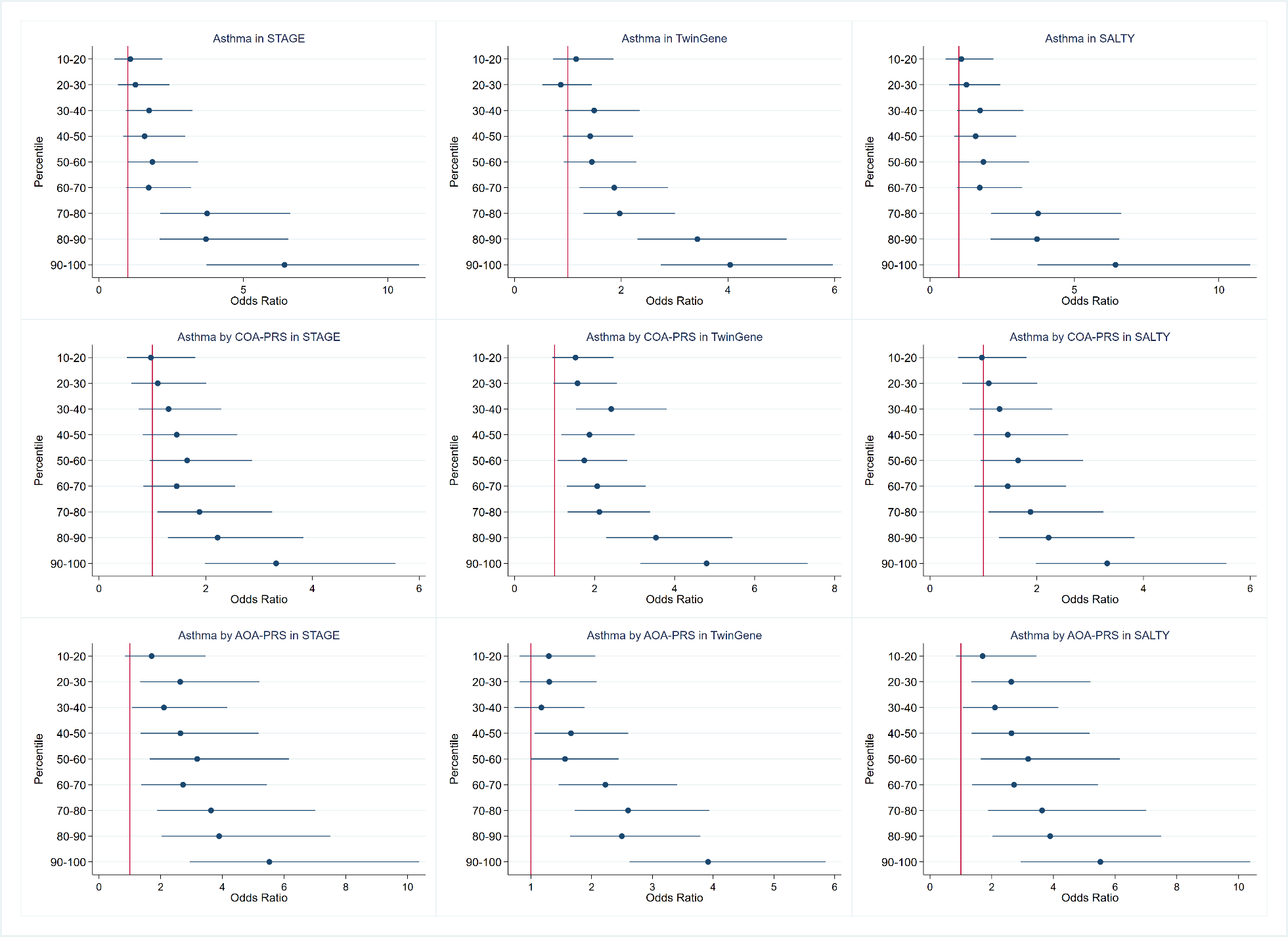


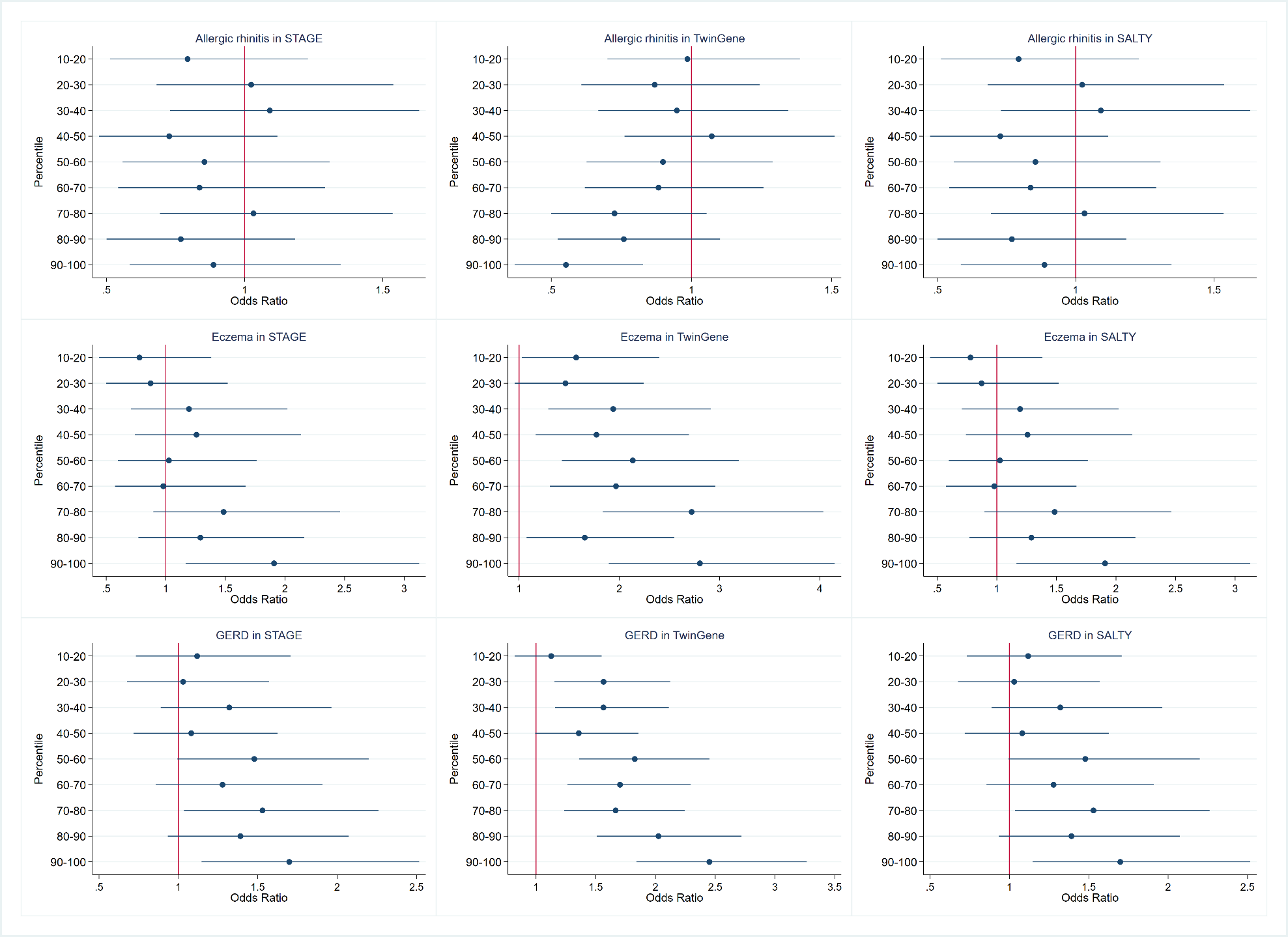


## **Supplementary Figure 3. Scatter plot and leave-one-out analysis for the association of asthma (instrumental variable, IV) with GERD (outcome)**

Figure legend.

Scatter plot: Estimated associations with GERD by asthma are plotted against predicted associations with the outcome from five MR tests (Inverse variance weighted, MR Egger, Simple mode, Weighed median and Weighed mode), presented in error bars with 95% confidence intervals.

Forest plot: Each horizontal error bar was an estimated genetic association with GERD (x-axis) by the rest asthma IVs with 95% CIs after omitting one specific IV indicated on the y axis.


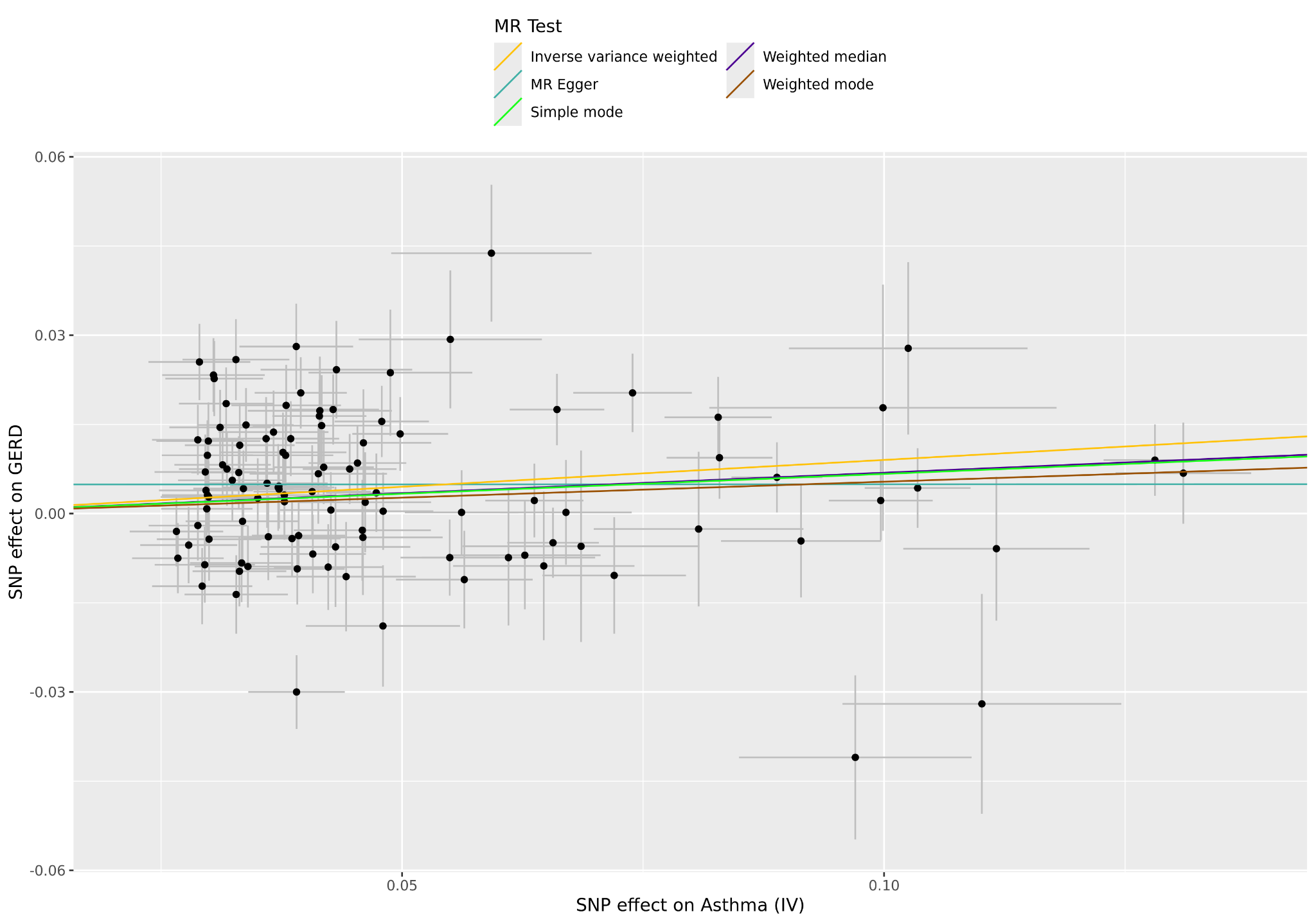


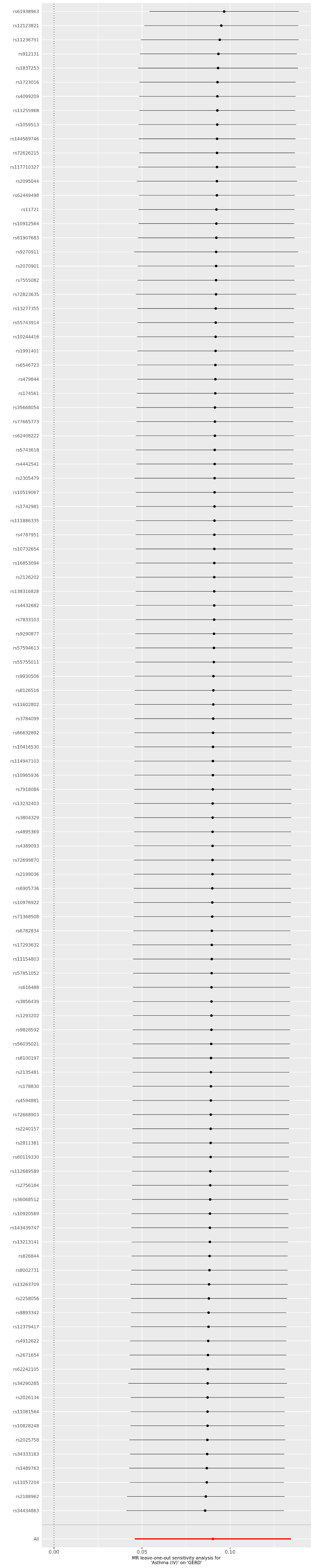


## **Supplementary Figure 4. Scatter plot and leave-one-out analysis for the association of GERD (IV) with asthma (outcome).**

Figure legend.

Scatter plot: Estimated associations with asthma by GERD are plotted against predicted associations with the outcome from five MR tests (Inverse variance weighted, MR Egger, Simple mode, Weighed median and Weighed mode), presented in error bars with 95% confidence intervals.

Forest plot: Each horizontal error bar was an estimated genetic association with asthma (x-axis) by the rest GERD IVs with 95% CIs after omitting one specific IV indicated on the y axis.


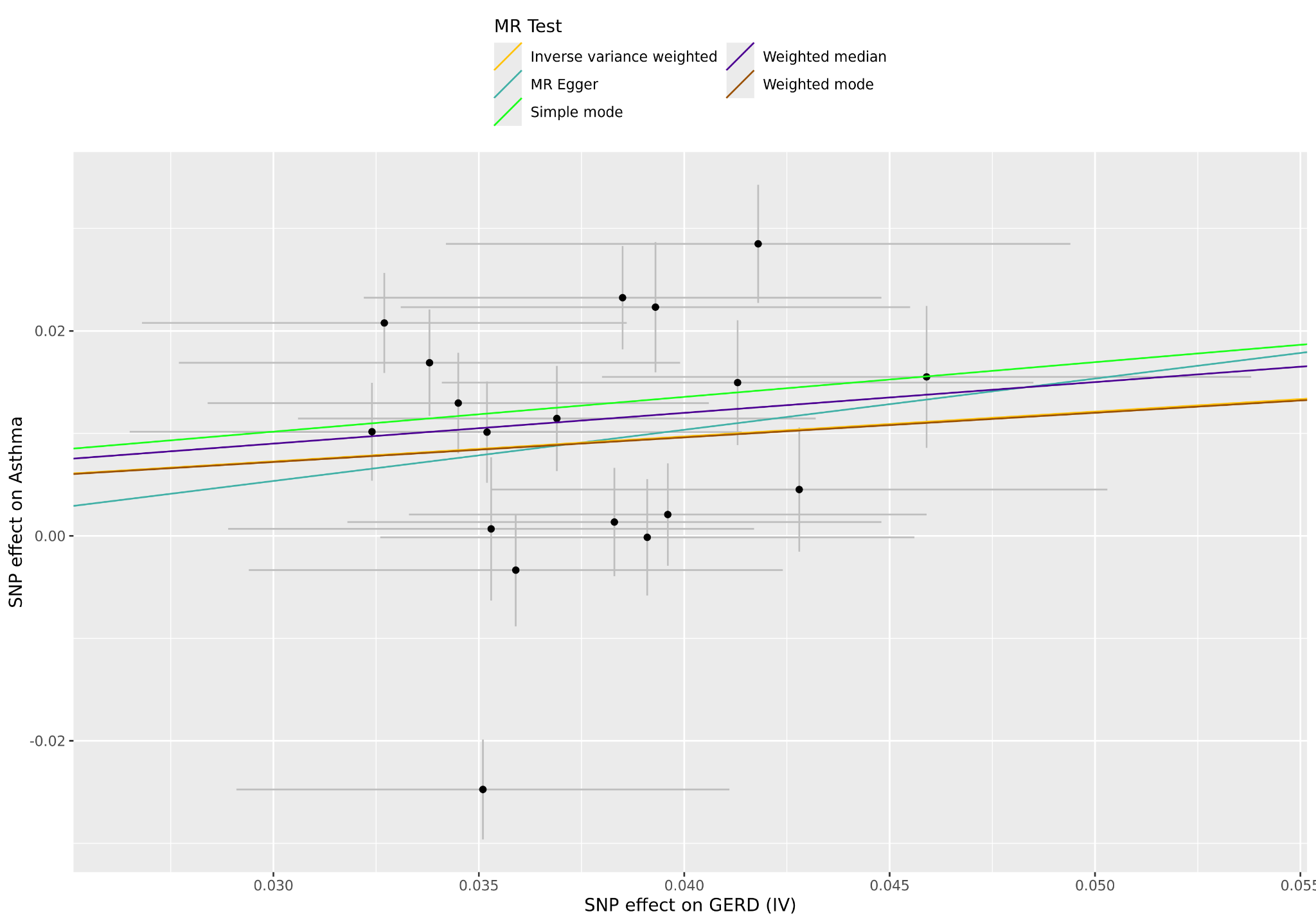


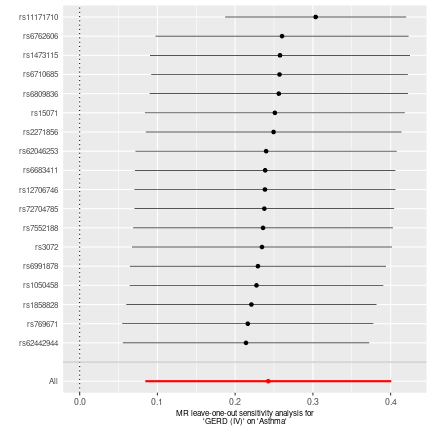


## **Supplementary Figure 5. Scatter plot and leave-one-out analysis for the association of eczema (IV) with GERD (outcome).**

Scatter plot: Estimated associations with GERD by eczema are plotted against predicted associations with the outcome from five MR tests (Inverse variance weighted, MR Egger, Simple mode, Weighed median and Weighed mode), presented in error bars with 95% confidence intervals.

Forest plot: Each horizontal error bar was an estimated genetic association with GERD (x-axis) by the rest eczema IVs with 95% CIs after omitting one specific IV indicated on the y axis.


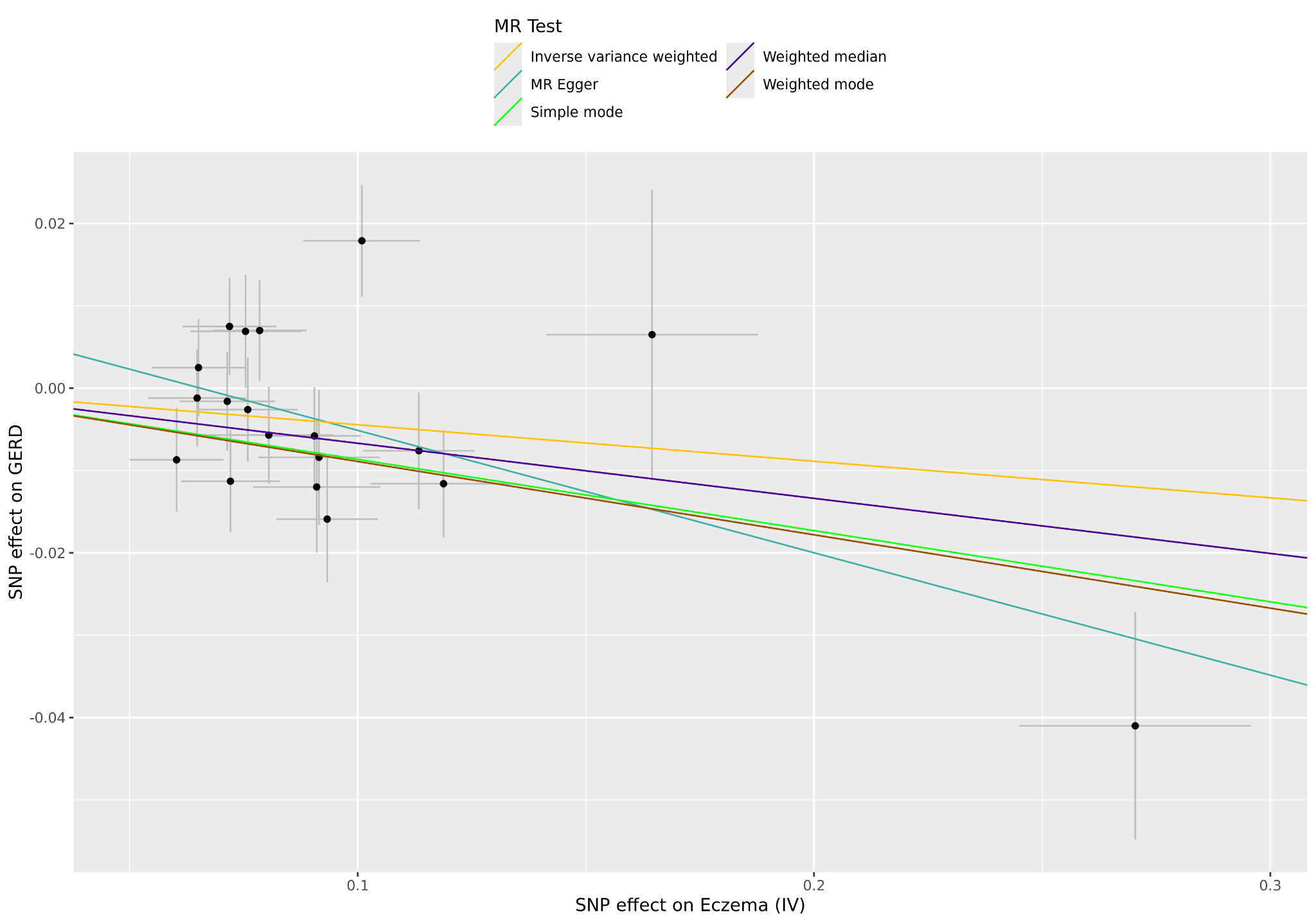


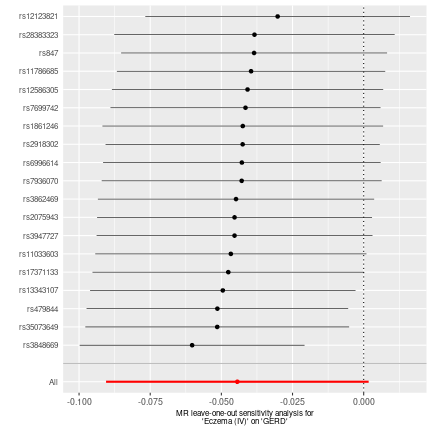


## **Supplementary Figure 6. Scatter plot and leave-one-out analysis for the association of allergic rhinitis (IV) with GERD (outcome).**

Scatter plot: Estimated associations with GERD by allergic rhinitis are plotted against predicted associations with the outcome from five MR tests (Inverse variance weighted, MR Egger, Simple mode, Weighed median and Weighed mode), presented in error bars with 95% confidence intervals.

Forest plot: Each horizontal error bar was an estimated genetic association with GERD (x-axis) by the rest allergic rhinitis IVs with 95% CIs after omitting one specific IV indicated on the y axis.


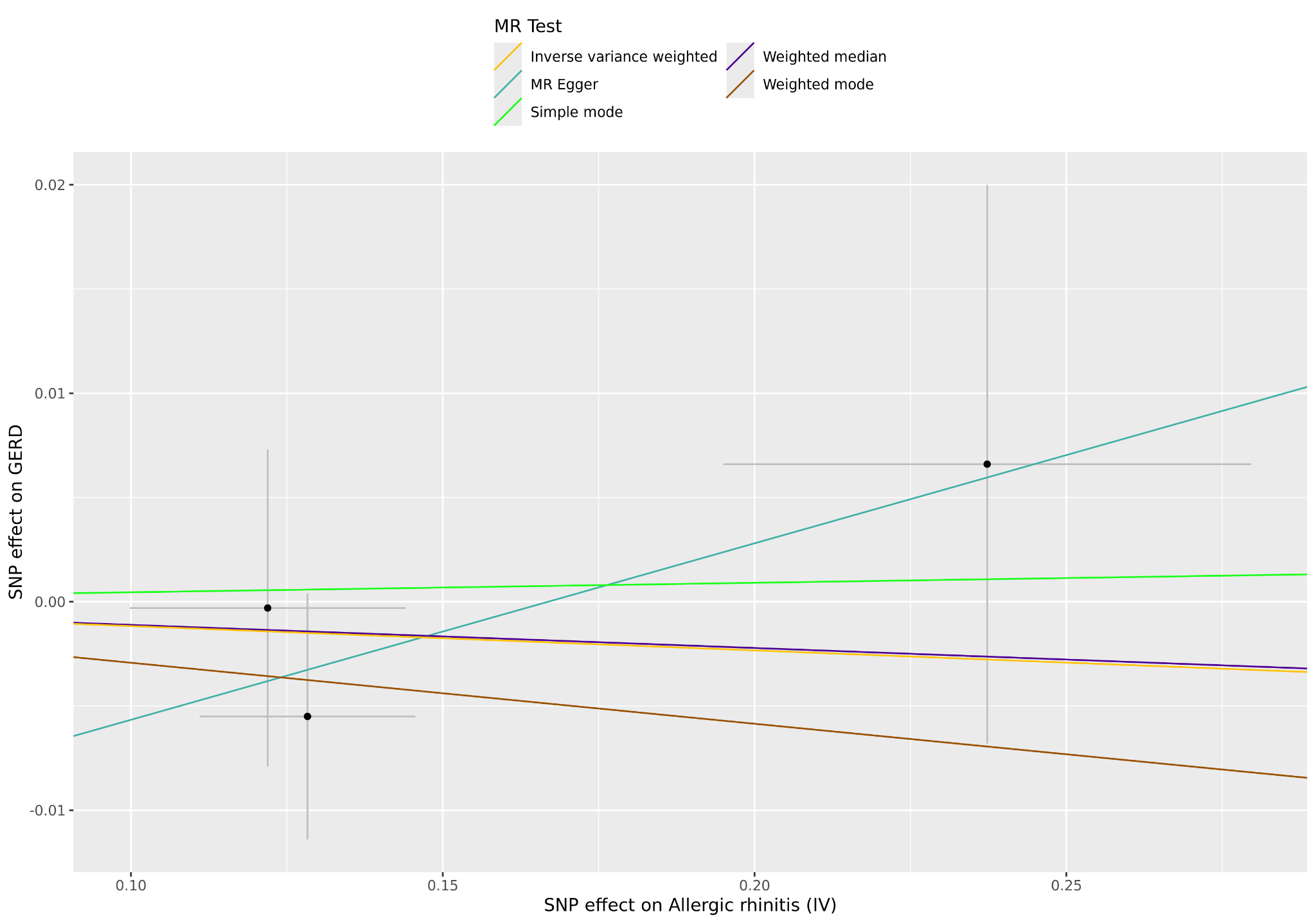


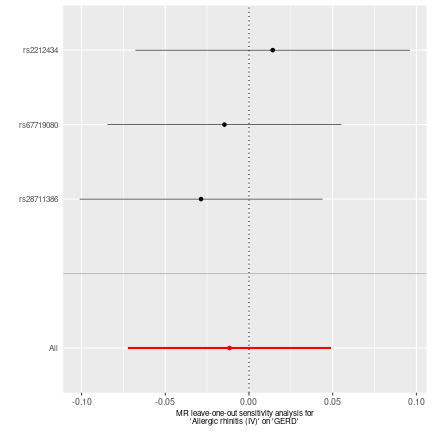


## **Supplementary Figure 7. Scatter plot and leave-one-out analysis for the association of GERD (IV) with allergic rhinitis (outcome).**

Scatter plot: Estimated associations with allergic rhinitis by GERD are plotted against predicted associations with the outcome from five MR tests (Inverse variance weighted, MR Egger, Simple mode, Weighed median and Weighed mode), presented in error bars with 95% confidence intervals.

Forest plot: Each horizontal error bar was an estimated genetic association with allergic rhinitis(x-axis) by the rest GERD IVs with 95% CIs after omitting one specific IV indicated on the y axis.


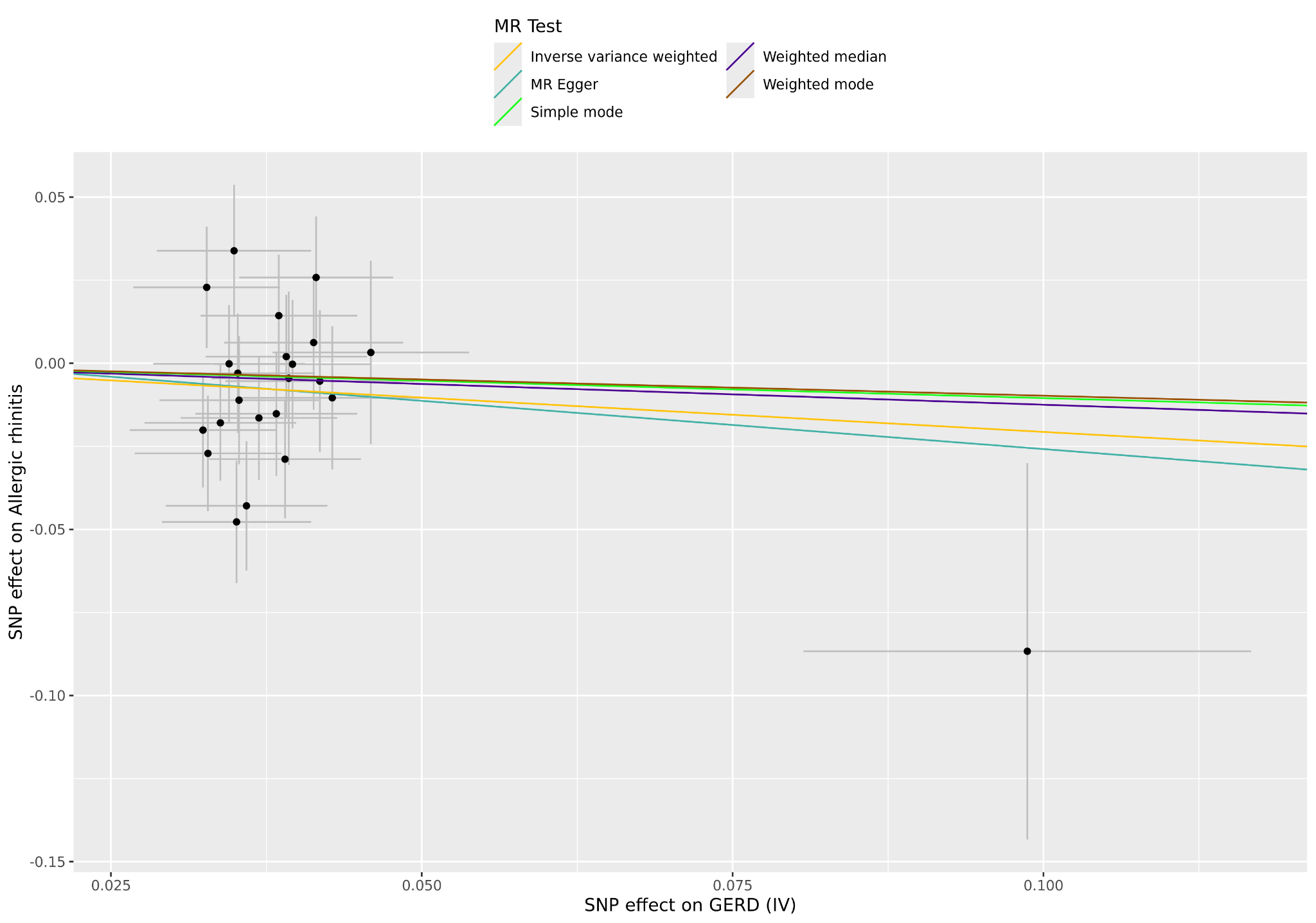


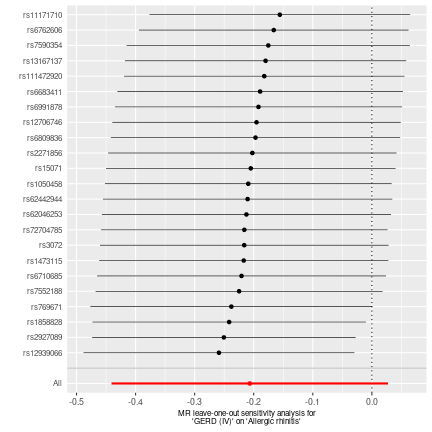


## **Supplementary Figure 8. Scatter plot and leave-one-out analysis for the association of GERD (IV) with eczema (outcome).**

Scatter plot: Estimated associations with eczema by GERD are plotted against predicted associations with the outcome from five MR tests (Inverse variance weighted, MR Egger, Simple mode, Weighed median and Weighed mode), presented in error bars with 95% confidence intervals.

Forest plot: Each horizontal error bar was an estimated genetic association with eczema (x-axis) by the rest GERD IVs with 95% CIs after omitting one specific IV indicated on the y axis.


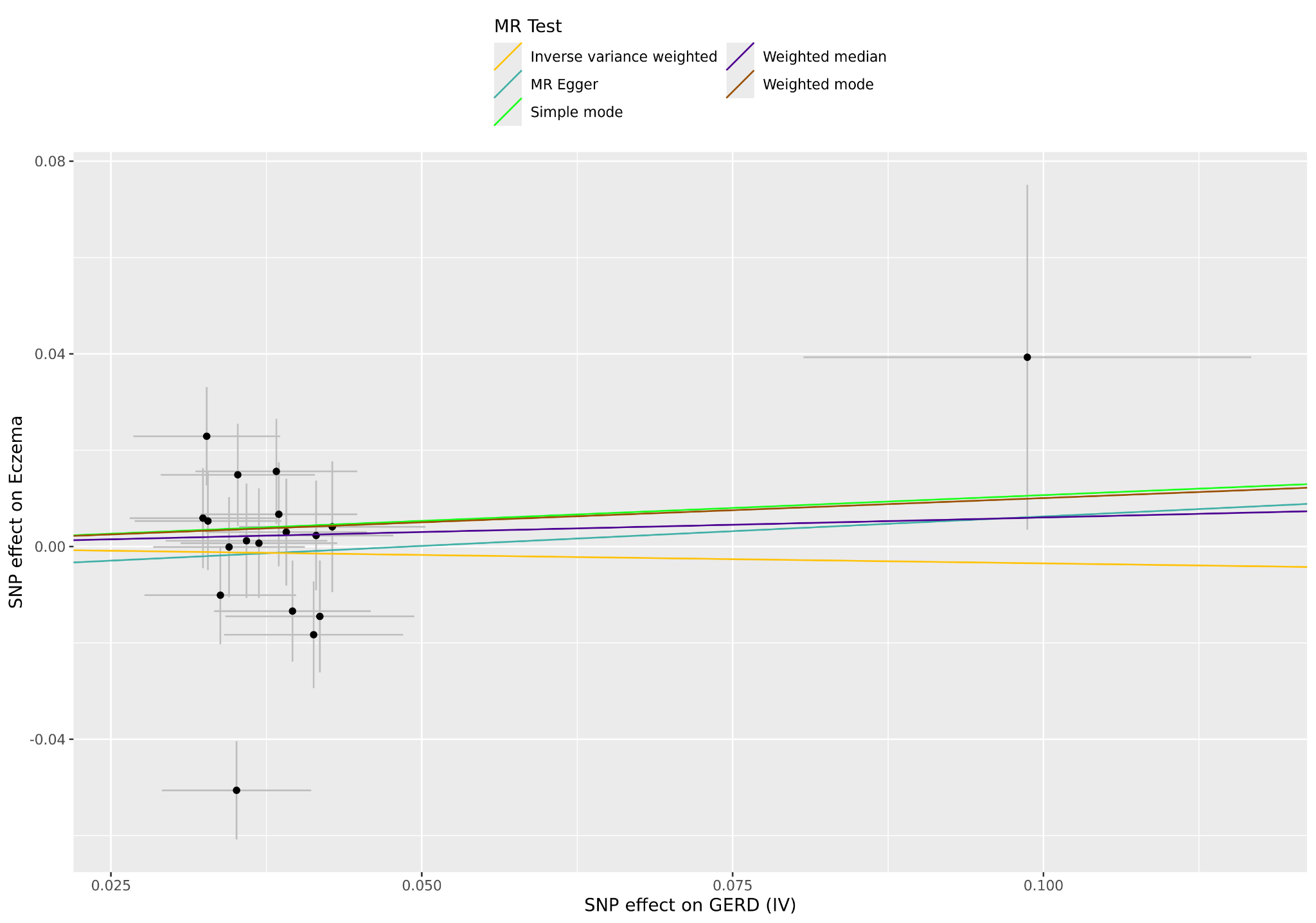


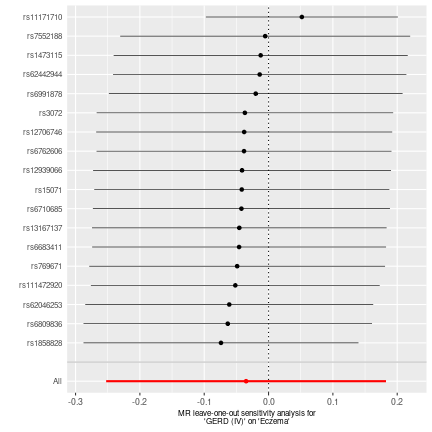


## **Supplementary Figure 9. Manhattan plot (gene-based test) for GERD.**

Figure Legend. Manhattan plot of the gene-based association test for GERD, estimated by MAGMA, as implemented on the FUMA platform. The input SNPs were mapped to 19119 protein coding genes. The red dash line indicates the genome wide significance defined at p = 0.05/19119 = 2.615 × 10–6.


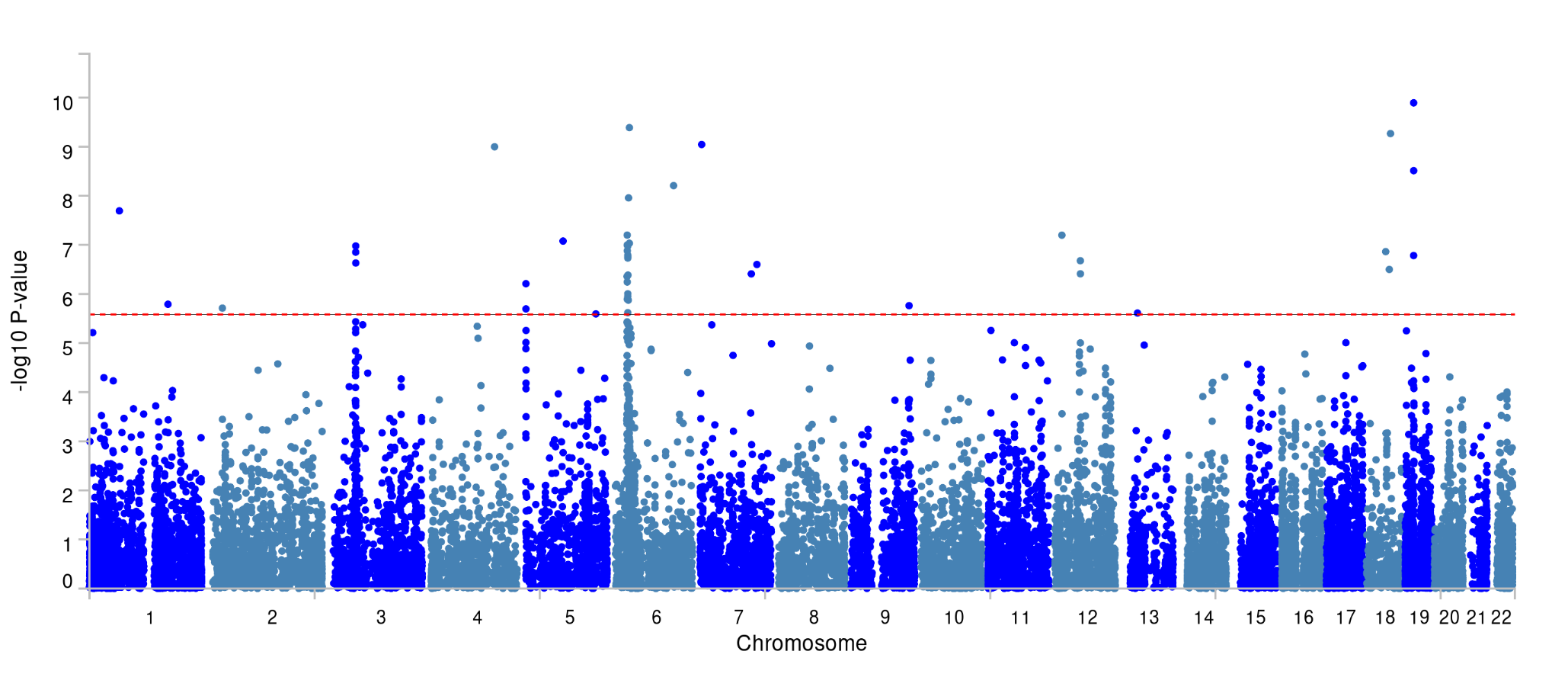


## **Supplementary Figure 10. Manhattan plot (gene-based test) for asthma.**

Figure Legend. Manhattan plot of the gene-based association test for asthma, estimated by MAGMA, as implemented on the FUMA platform. The input SNPs were mapped to 19119 protein coding genes. The red dash line indicates the genome wide significance defined at p = 0.05/19119 = 2.615 × 10–6.


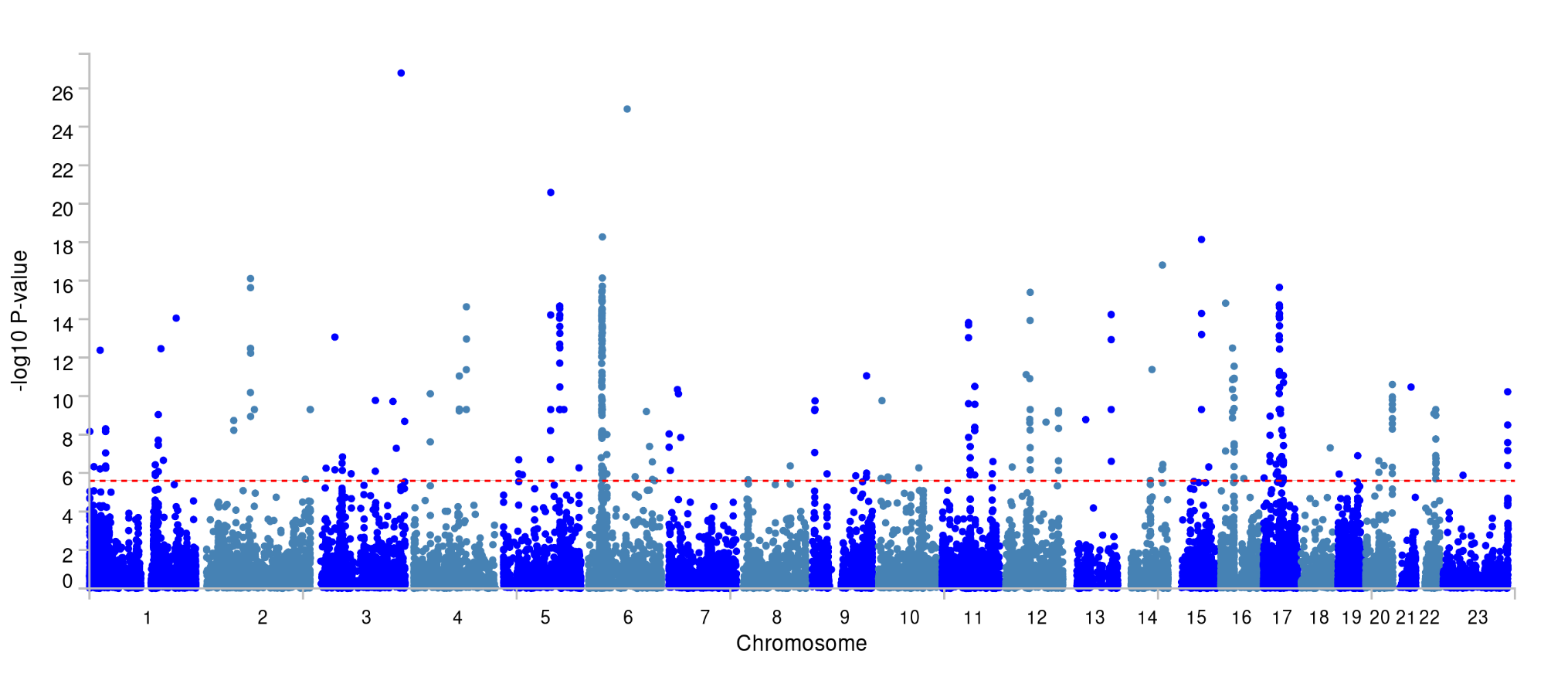


## **Supplementary Figure 11. Manhattan plot (gene-based test) for allergic rhinitis.**

Figure Legend. Manhattan plot of the gene-based association test for allergic rhinitis, estimated by MAGMA, as implemented on the FUMA platform. The input SNPs were mapped to 19119 protein coding genes. The red dash line indicates the genome wide significance defined at p = 0.05/19119 = 2.615 × 10–6.


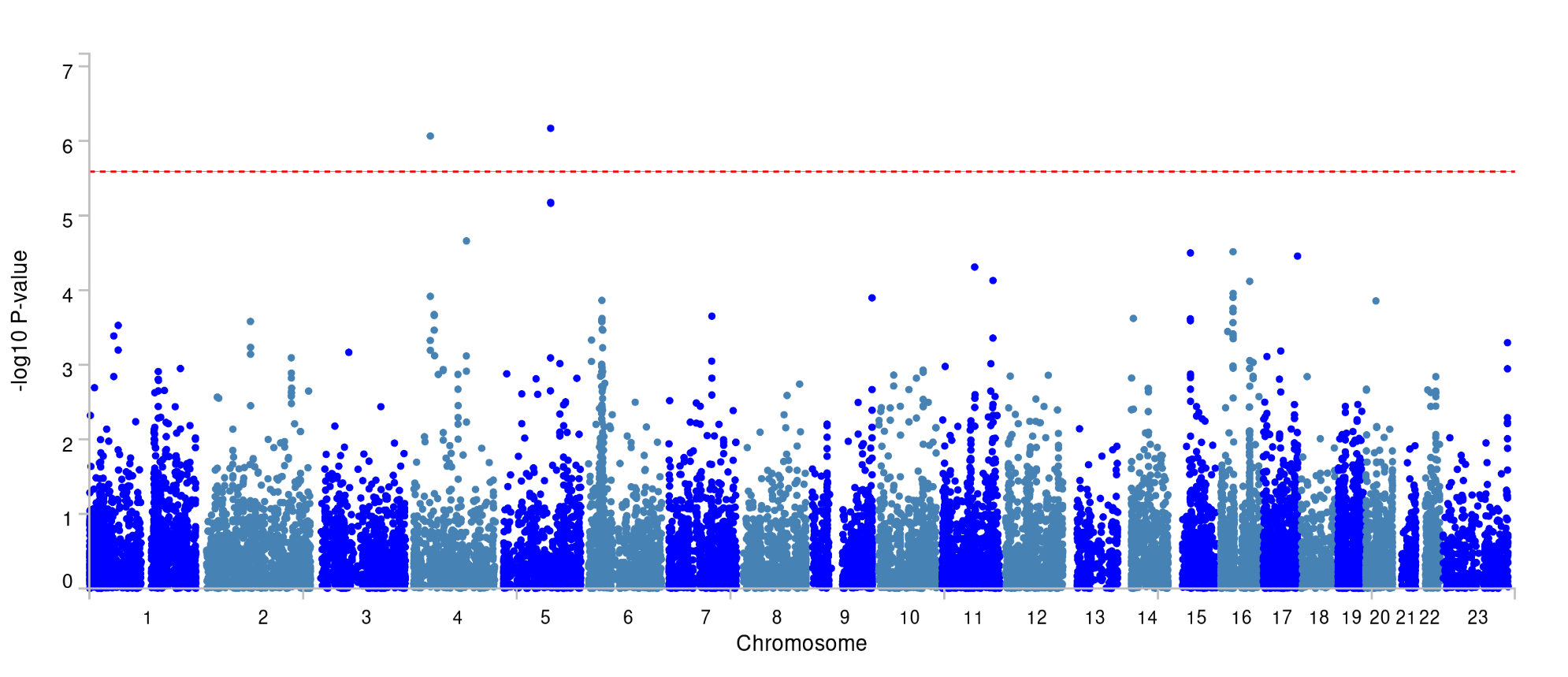


## **Supplementary Figure 12. Manhattan plot (gene-based test) for eczema**

Figure Legend. Manhattan plot of the gene-based association test for eczema, estimated by MAGMA, as implemented on the FUMA platform. The input SNPs were mapped to 19119 protein coding genes. The red dash line indicates the genome wide significance defined at p = 0.05/19119 = 2.615 × 10–6.


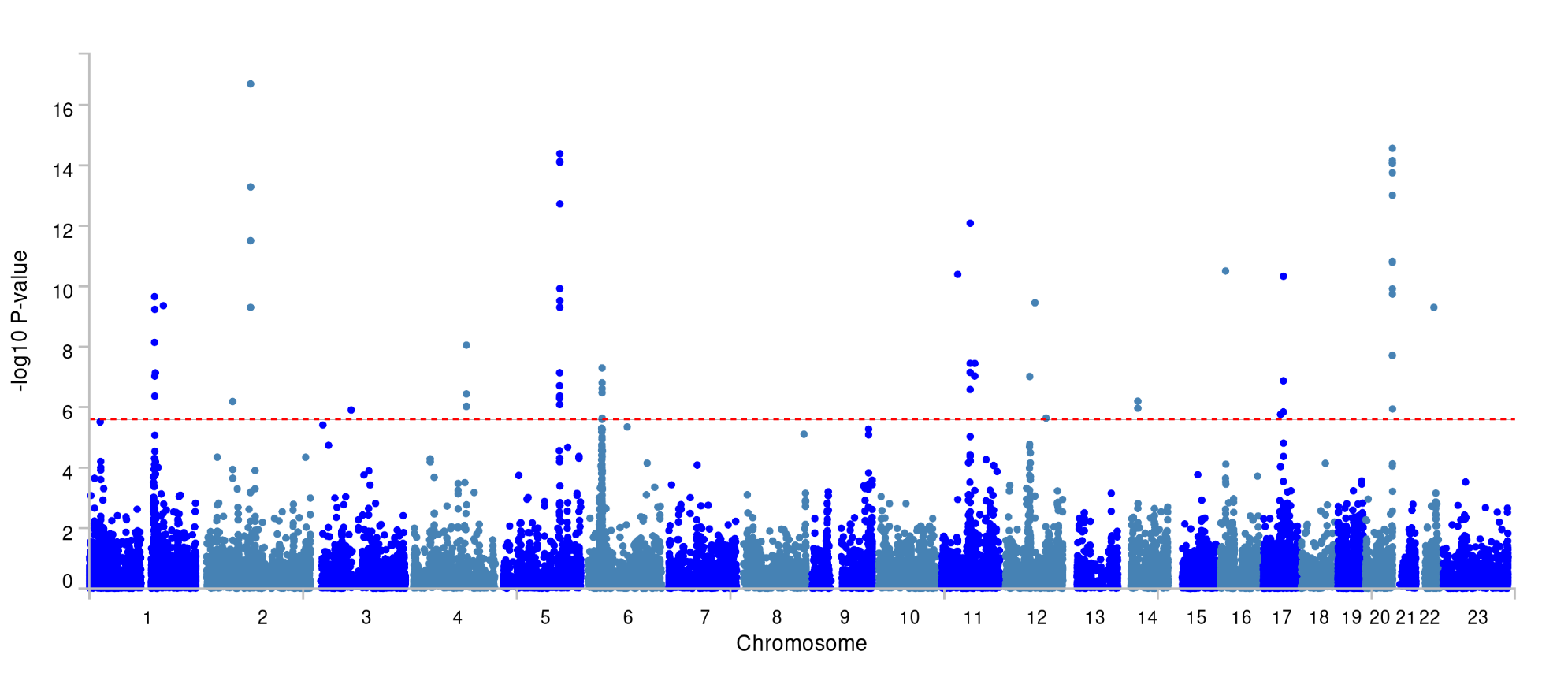


## **Supplementary Figure 13. Regional association plot for asthma (up) and GERD (down) close to RAB5B and ERBB3 genes.**


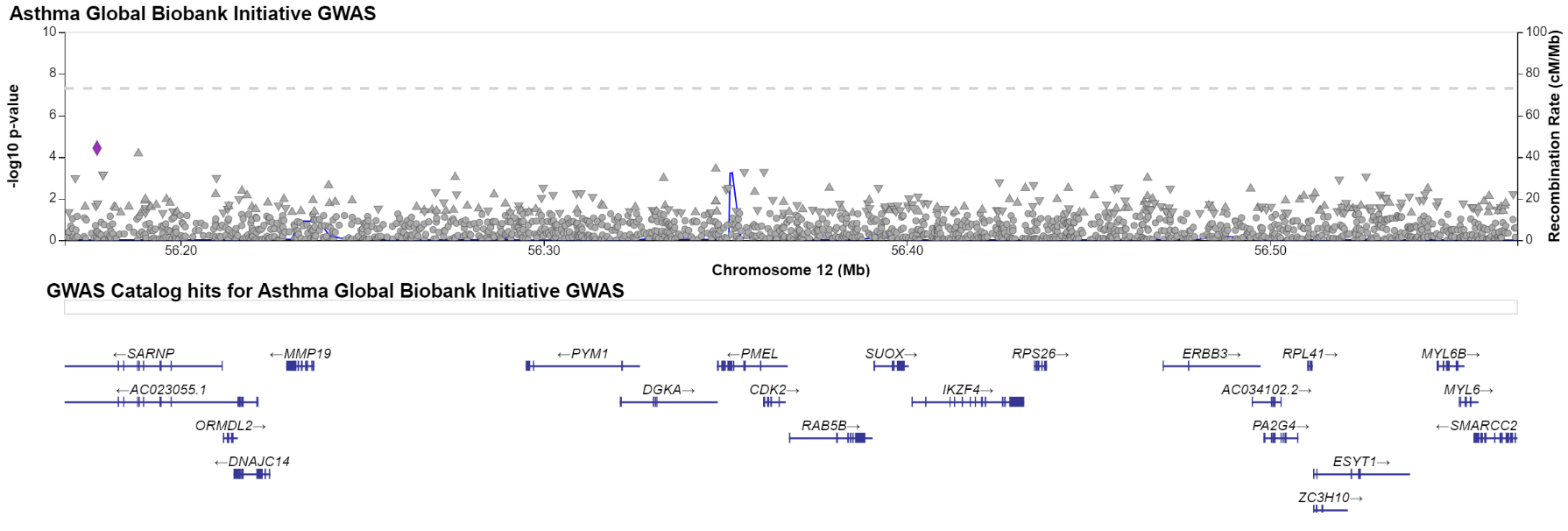

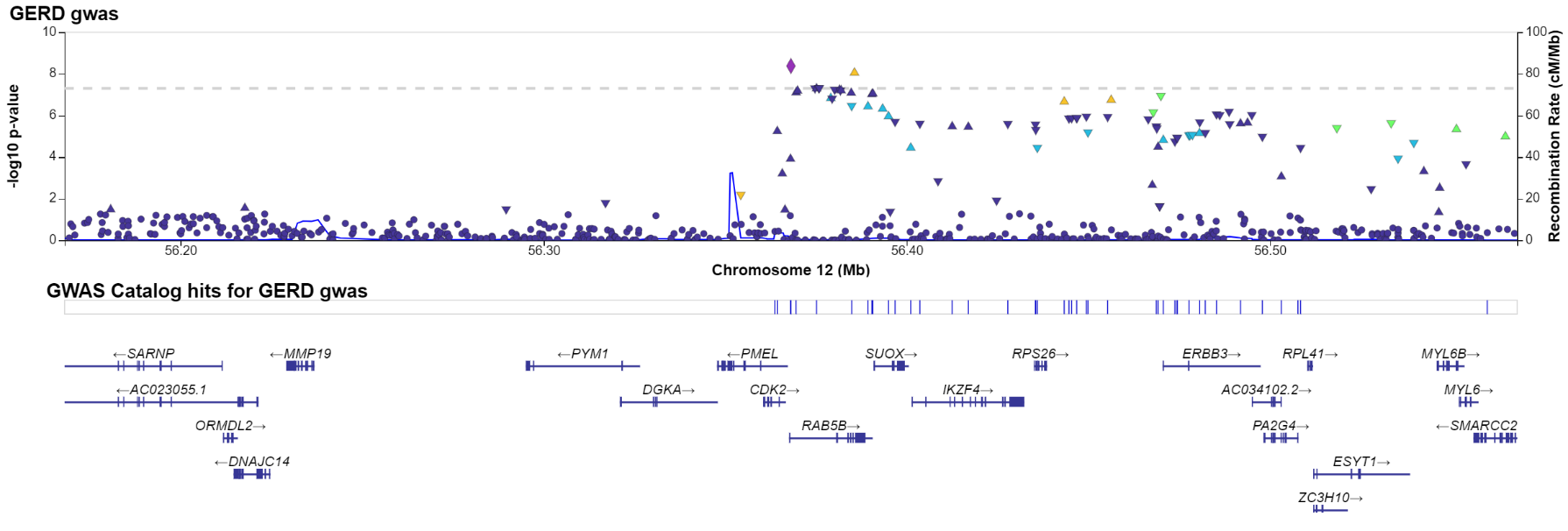


## **Supplementary Figure 14. Regional association plot for asthma (up) and GERD (down) close to RBM6 gene.**


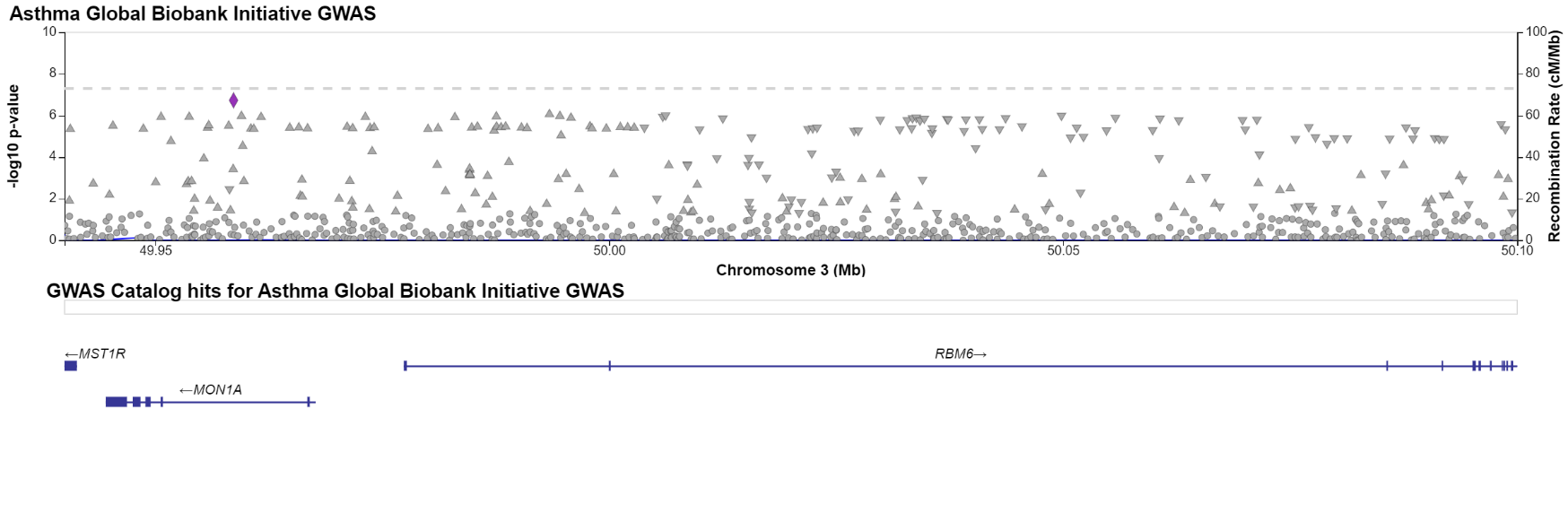


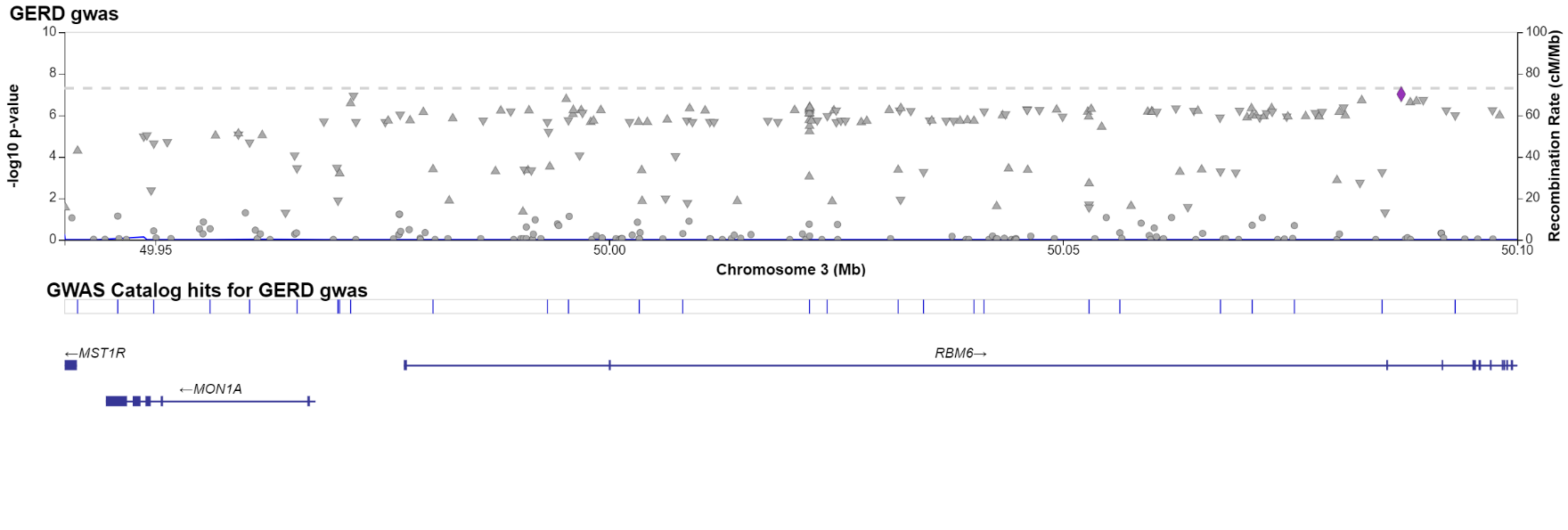


## **Supplementary Figure 15. Regional association plot for asthma (up) and GERD (down) close to SDK1 gene.**


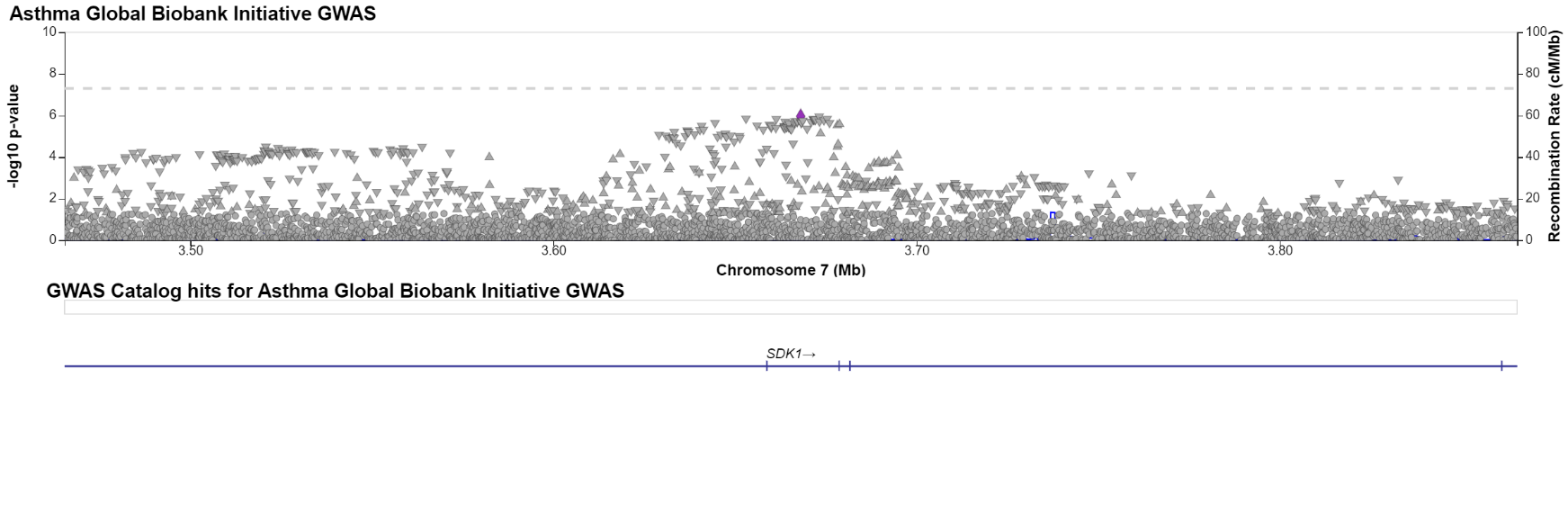

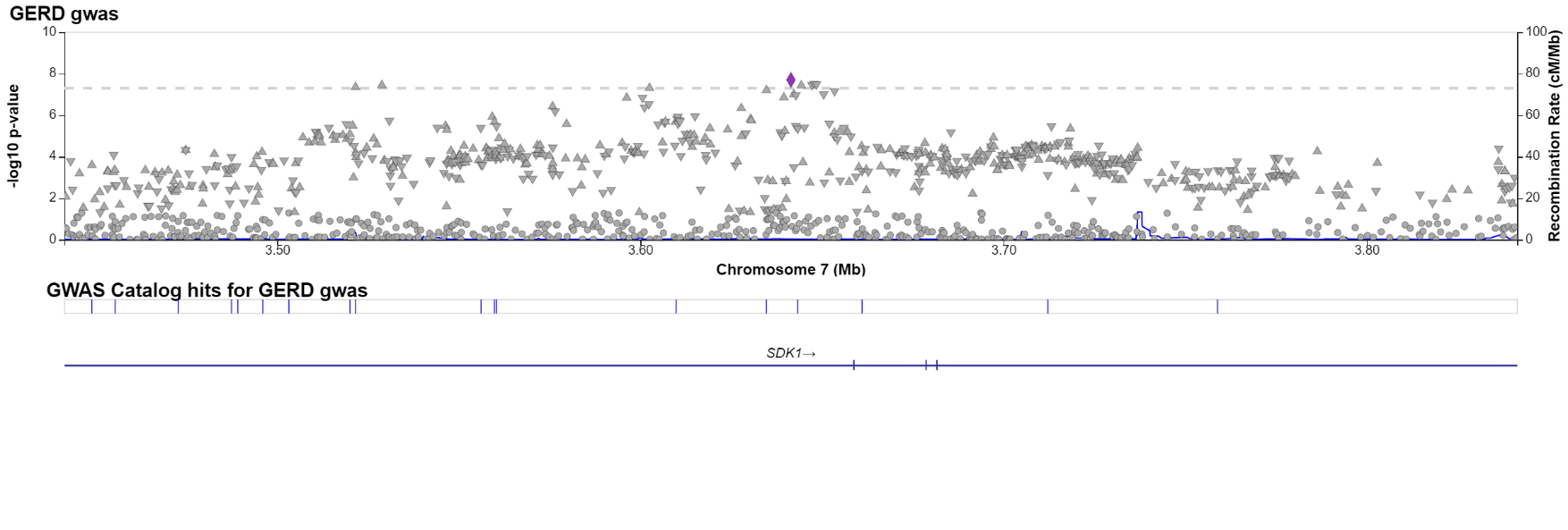


## **Supplementary Figure 16. Regional association plot for asthma (up) and GERD (down) close to HLA-B gene.**


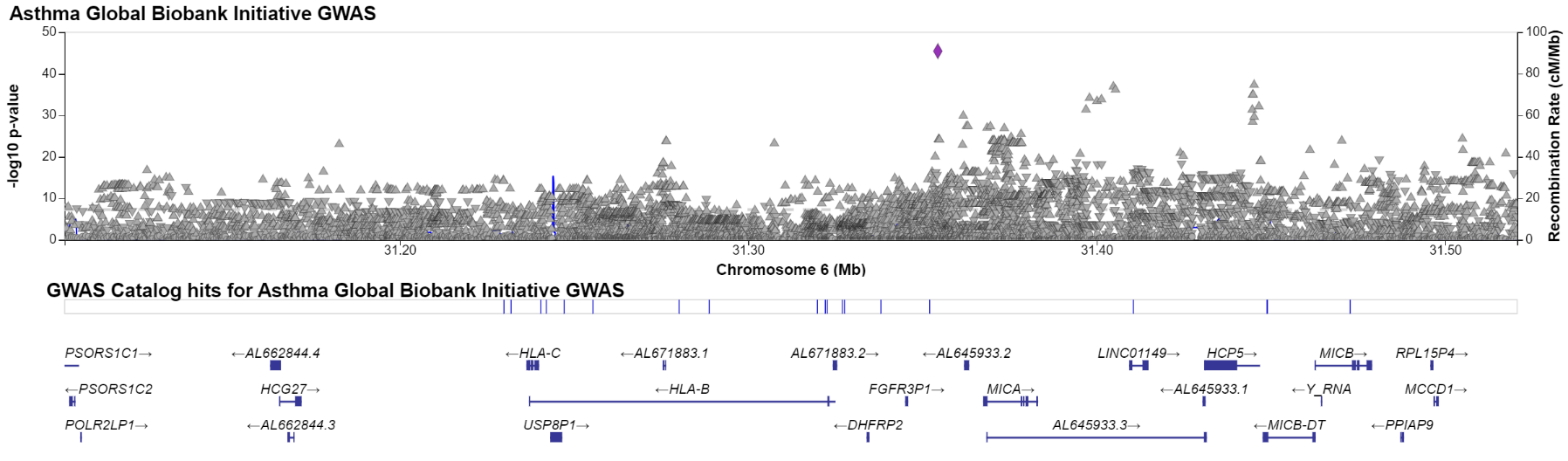


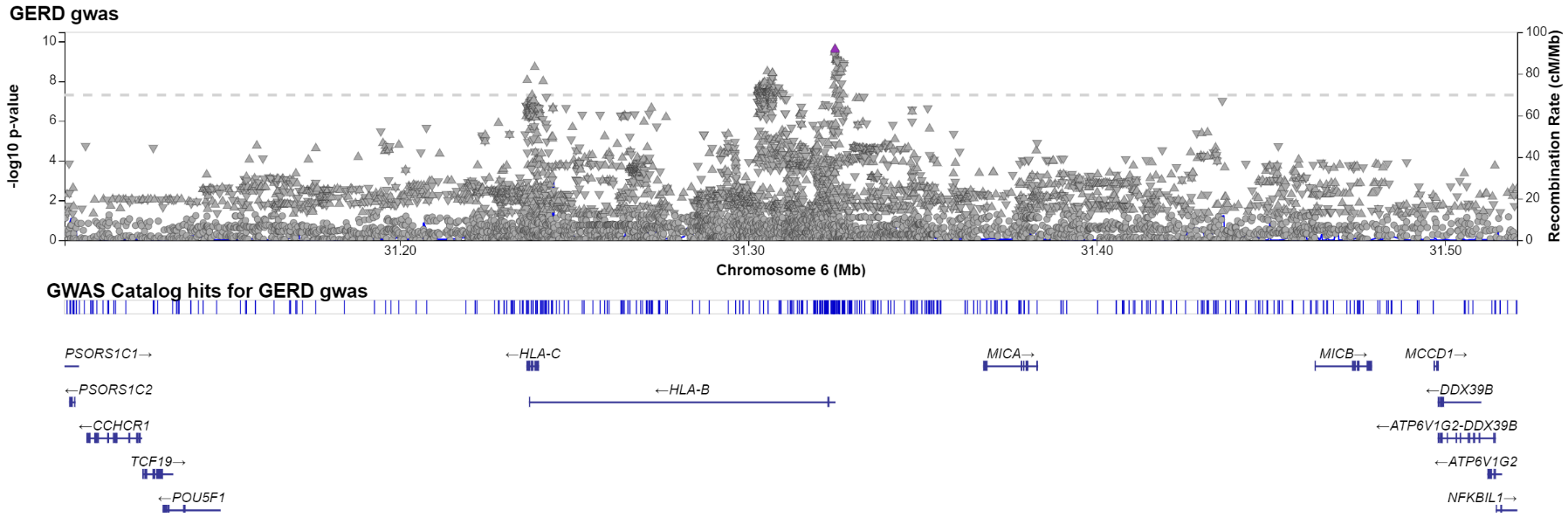


## **Supplementary Figure 17. Regional association plot for asthma (up) and GERD (down) close to RERG gene.**


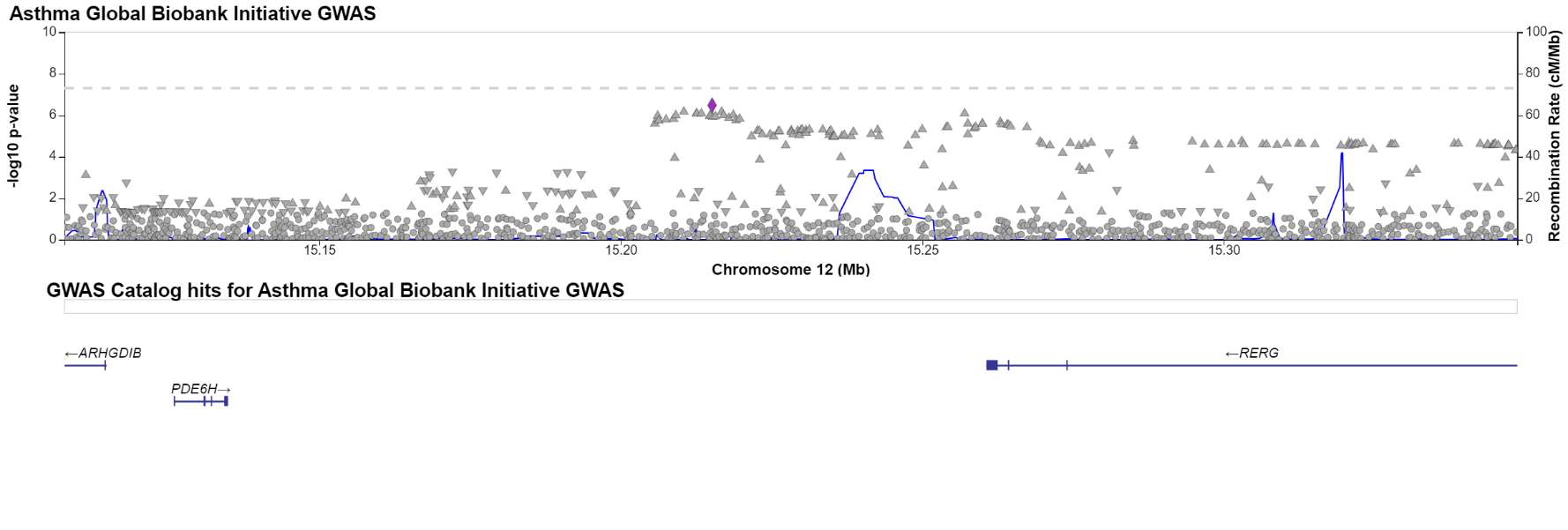


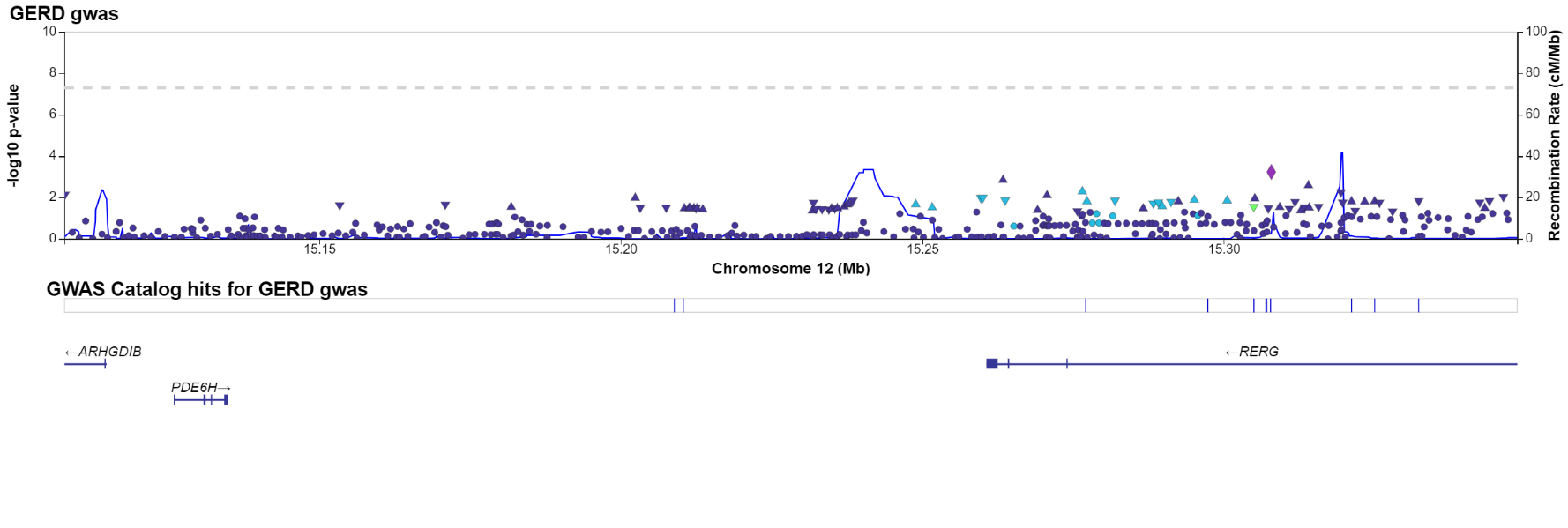


## **Supplementary Figure 18. MAGMA Tissue Expression Analysis of GERD and plotting differentially expressed genes in relevant tissues (GTEx v8 30 general tissue types).**

Legend. Overview of the results of the MAGMA gene-level tissue enrichment analysis of GERD, as implemented on the FUMA platform, using GTEx v8 data for 30 general tissue types. Nominal −log10 p-values are shown on the y-axis. Brain tissues showed overall significant enrichment after correction for multiple testing (in red bar) but no differential gene expression signals were detected.


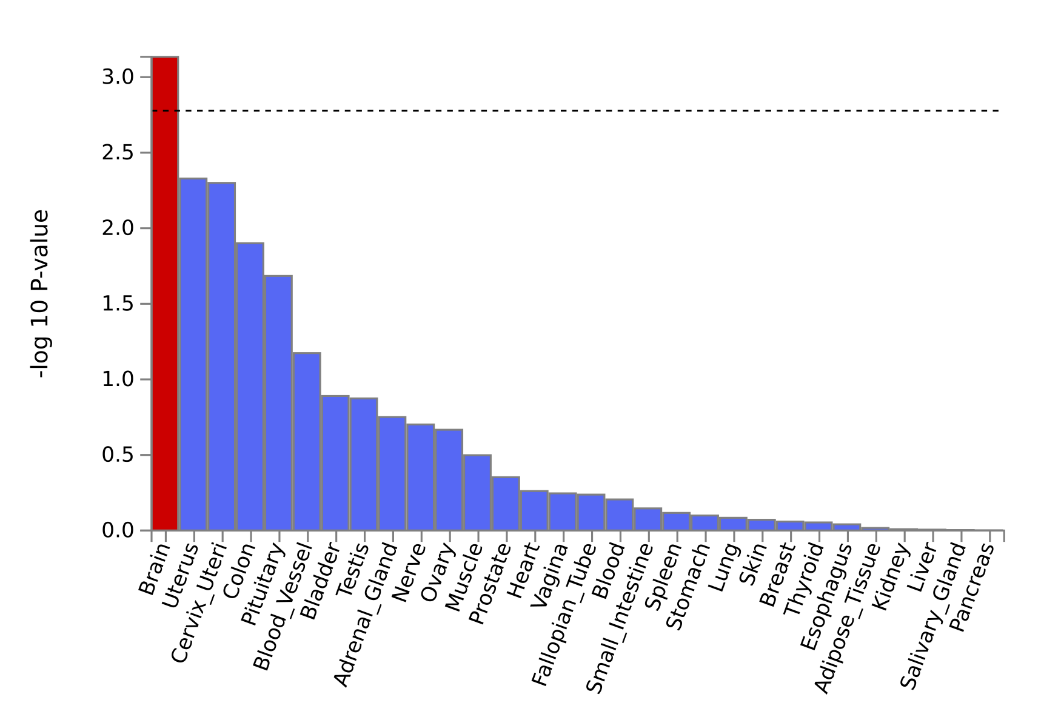

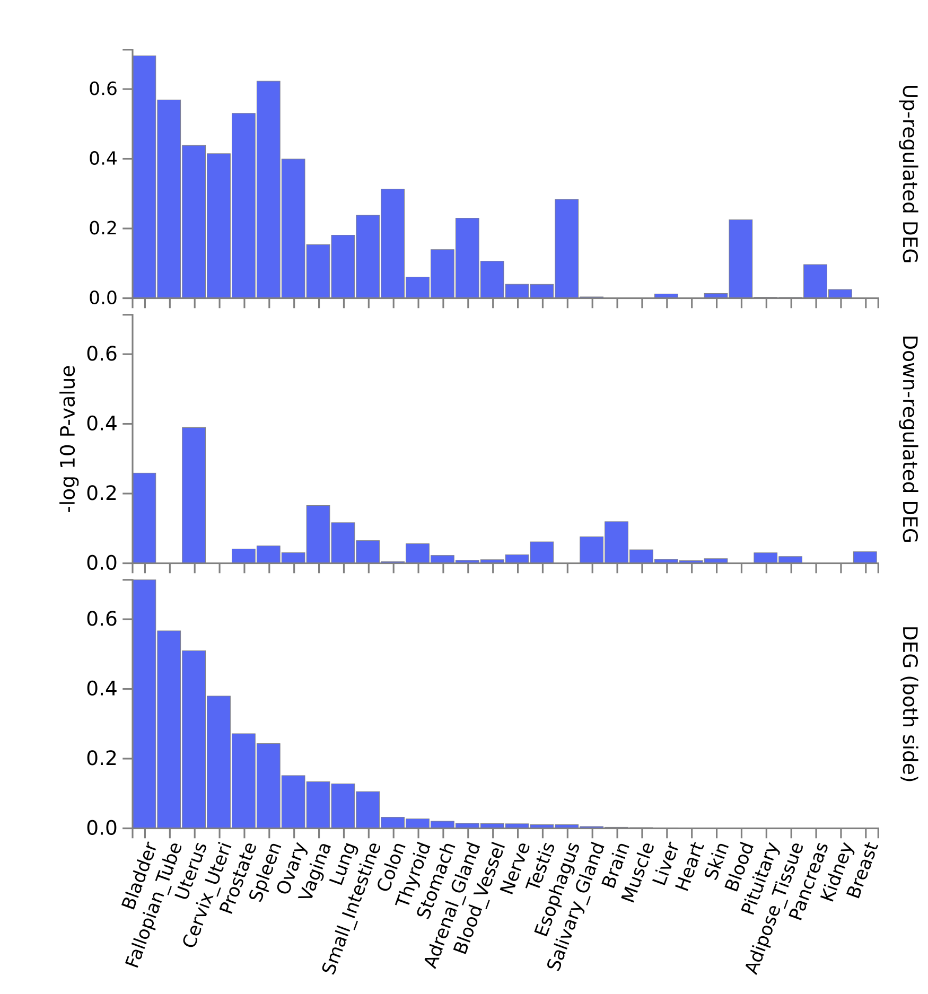


## **Supplementary Figure 19. MAGMA Tissue Expression Analysis of asthma and plotting differentially expressed genes in relevant tissues (GTEx v8 30 general tissue types)**

Legend. Overview of the results of the MAGMA gene-level tissue enrichment analysis of asthma, as implemented on the FUMA platform, using GTEx v8 data for 30 general tissue types. Nominal −log10 p-values are shown on the y-axis. Blood, spleen, lung and small intestine tissues showed overall significant enrichment after correction for multiple testing (in red bars). The mapped genes were significantly upregulated in blood, lung, and small intestine.


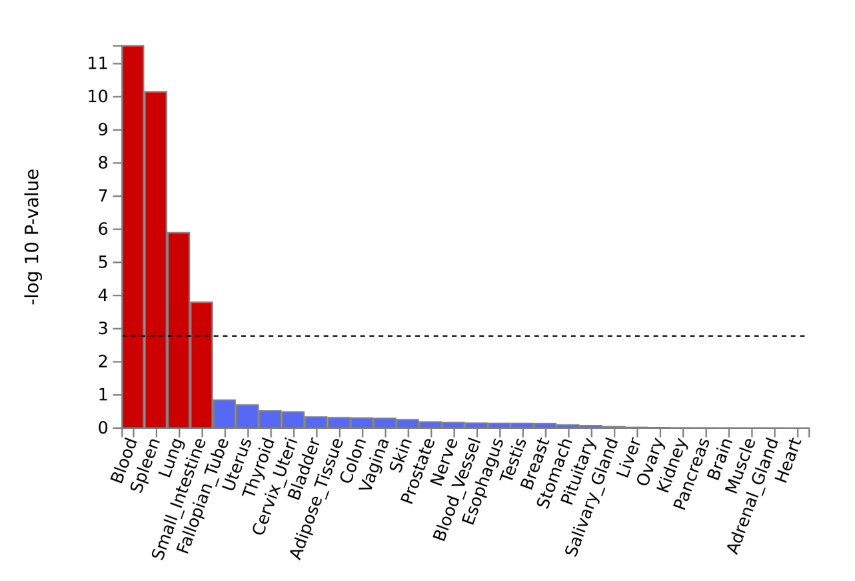

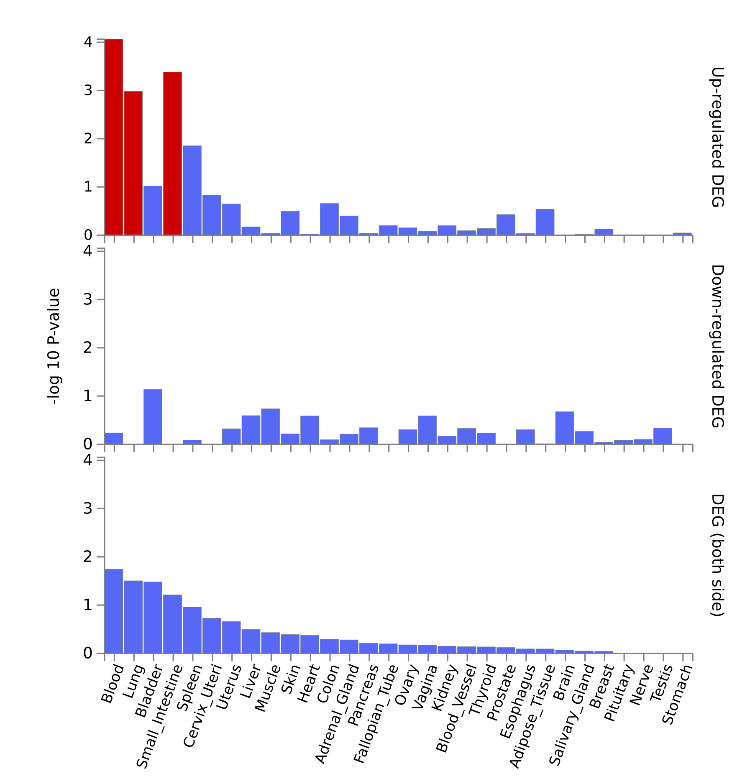


## **Supplementary Figure 20. MAGMA Tissue Expression Analysis of allergic rhinitis and plotting differentially expressed genes in relevant tissues (GTEx v8 30 general tissue types)**

Legend. Overview of the results of the MAGMA gene-level tissue enrichment analysis of asthma, as implemented on the FUMA platform, using GTEx v8 data for 30 general tissue types. Nominal −log10 p-values are shown on the y-axis. None of the tissues showed enrichment signals after correction for multiple testing.


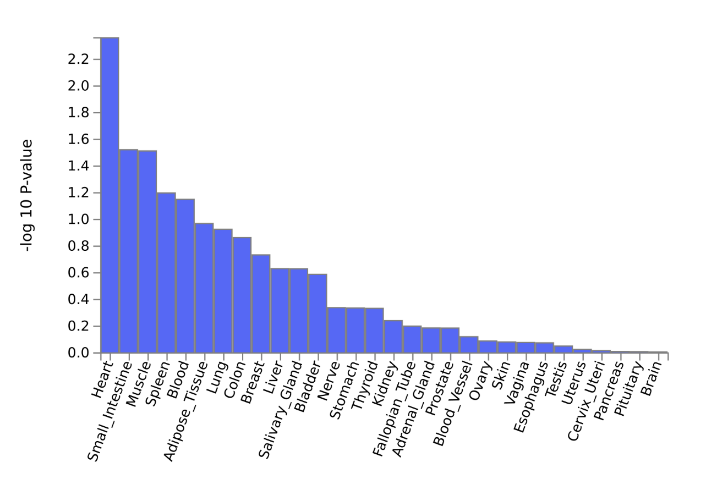

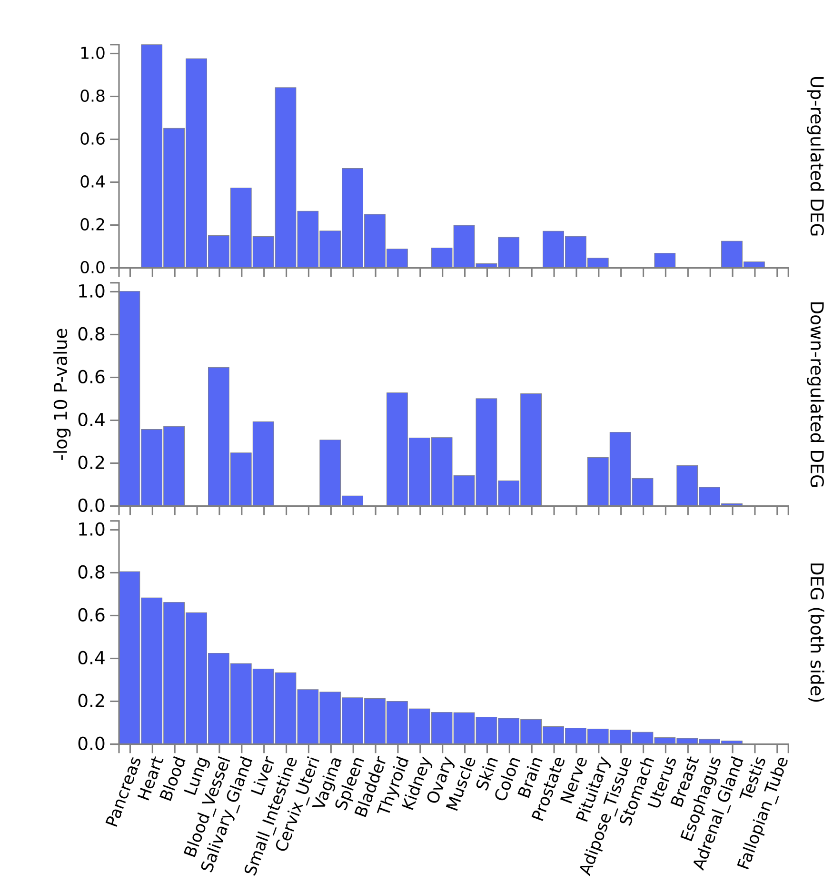


## **Supplementary Figure 21. MAGMA Tissue Expression Analysis of eczema and plotting differentially expressed genes in relevant tissues (GTEx v8 30 general tissue types)**

Legend. Overview of the results of the MAGMA gene-level tissue enrichment analysis of asthma, as implemented on the FUMA platform, using GTEx v8 data for 30 general tissue types. Nominal −log10 p-values are shown on the y-axis. Spleen, blood and small intestine tissues showed overall significant enrichment after correction for multiple testing (in red bars). The mapped genes were significantly upregulated in skin.


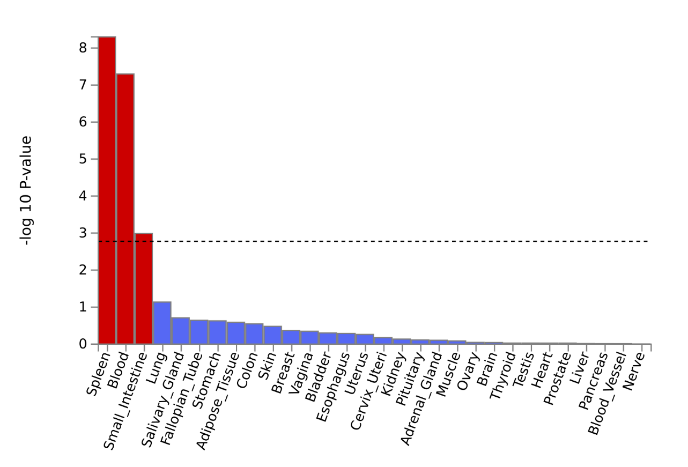

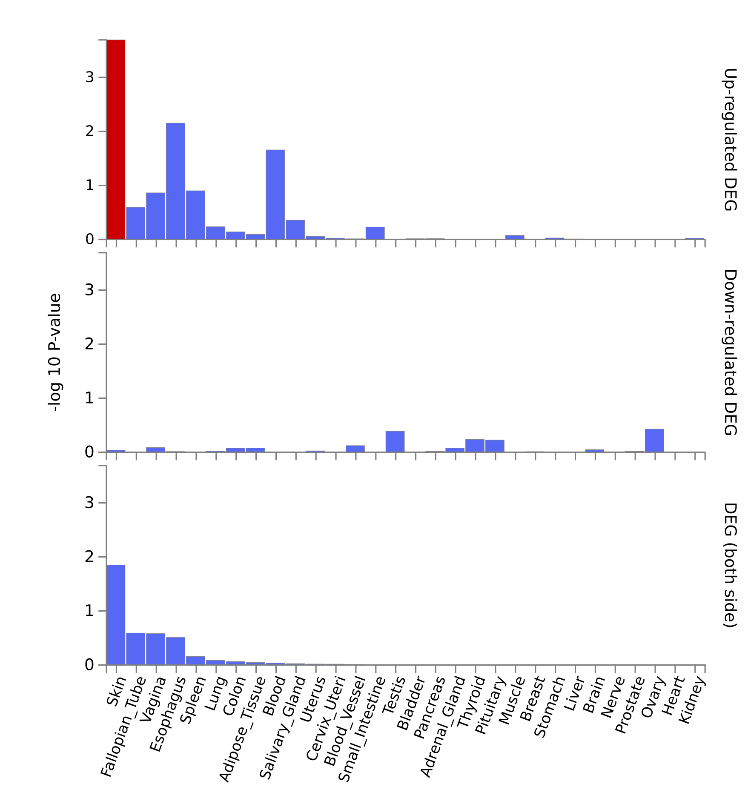

Supplement: Supplementary file 2 — Supplementary Information [file 42003_2024_6795_MOESM2_ESM.docx]
