# Supplementary material for: Shared genetic architecture between gastro-esophageal reflux disease, asthma, and allergic diseases
Source: Commun Biol. 2024 Sep 2;7:1077. doi: 10.1038/s42003-024-06795-1 (PMC11369275; doi:10.1038/s42003-024-06795-1)
Supplement: Supplementary file 3 — Reporting summary [file 42003_2024_6795_MOESM3_ESM.pdf]

nature portfolio

Corresponding author(s): Bronwyn K Brew

Last updated by author(s): Aug 6, 2024

Reporting Summary

Nature Portfolio wishes to improve the reproducibility of the work that we publish. This form provides structure for consistency and transparency in reporting. For further information on Nature Portfolio policies, see our Editorial Policies and the Editorial Policy Checklist.

Statistics

For all statistical analyses, confirm that the following items are present in the figure legend, table legend, main text, or Methods section.

n/a	Confirmed	
☐	☒	The exact sample size (*n*) for each experimental group/condition, given as a discrete number and unit of measurement
☐	☒	A statement on whether measurements were taken from distinct samples or whether the same sample was measured repeatedly
☐	☒	The statistical test(s) used AND whether they are one- or two-sided<br>*Only common tests should be described solely by name; describe more complex techniques in the Methods section.*
☐	☒	A description of all covariates tested
☐	☒	A description of any assumptions or corrections, such as tests of normality and adjustment for multiple comparisons
☐	☒	A full description of the statistical parameters including central tendency (e.g. means) or other basic estimates (e.g. regression coefficient) AND variation (e.g. standard deviation) or associated estimates of uncertainty (e.g. confidence intervals)
☐	☒	For null hypothesis testing, the test statistic (e.g. *F*, *t*, *r*) with confidence intervals, effect sizes, degrees of freedom and *P* value noted<br>*Give P values as exact values whenever suitable.*
☒	☐	For Bayesian analysis, information on the choice of priors and Markov chain Monte Carlo settings
☒	☐	For hierarchical and complex designs, identification of the appropriate level for tests and full reporting of outcomes
☐	☒	Estimates of effect sizes (e.g. Cohen's *d*, Pearson's *r*), indicating how they were calculated

Our web collection on statistics for biologists contains articles on many of the points above.

Software and code

Policy information about availability of computer code

Data collection	Swedish Twin Register and published GWAS summary statistics for asthma, allergic rhinitis, eczema and gastro-esophageal reflux disease
Data analysis	Quantitative genetic analyses were performed in R (version 3.6.2) with the OpenMx package (version 2.14.1) using custom code. Polygenic risk score analysis used SAS 9.4 and custom code. Linkage disequilibirum score regression used published GWAS summary statistics and analysis used the LDSC command line tool in Python 2.7.5. Gene-based association analysis utlised GWAS summary statistics using MAGMA v1.6 on the FUMA platform. Mendelian Randomization and Genomic SEM were performed in R (version 4.2.3).

For manuscripts utilizing custom algorithms or software that are central to the research but not yet described in published literature, software must be made available to editors and reviewers. We strongly encourage code deposition in a community repository (e.g. GitHub). See the Nature Portfolio guidelines for submitting code & software for further information.

Data

Policy information about availability of data

All manuscripts must include a data availability statement. This statement should provide the following information, where applicable:

- Accession codes, unique identifiers, or web links for publicly available datasets
- A description of any restrictions on data availability
- For clinical datasets or third party data, please ensure that the statement adheres to our policy

Original data are held by Swedish National Board of Health and Welfare, Statistics Sweden and the Swedish Twin Registry, https://ki.se/en/research/the-swedish-

twin-registry. Due to Swedish data storage laws we cannot make the data publicly available, however, any researcher can access the data by obtaining an ethical approval and then asking the registers for the original data. Pseudonymised data may also be provided from the PI upon requests if providing a reasonable proposal and if an appropriate data sharing agreement with Karolinska Institutet can be established.
Published GWAS summary statistics were sourced from: https://genepi.qimr.edu.au/staff/manuelF/gwas_results/CHILD_ONSET_ASTHMA.20180501.allchr.assoc.GC.gz , https://genepi.qimr.edu.au/staff/manuelF/gwas_results/ADULT1_ADULT2_ONSET_ASTHMA.20180716.allchr.assoc.GC.gz, https://github.com/globalbiobank , https://hmgubox.helmholtz-muenchen.de/d/b55da086360c40118ae8/files/?p=/2018-05-11_EAGLE_AR.txt.gz, http://ftp.ebi.ac.uk/pub/databases/gwas/summary_statistics/GCST90027001-GCST90028000/GCST90027161/harmonised/34454985-GCST90027161-EFO_0000274.h.tsv.gz, https://figshare.com/articles/dataset/GERD_GWAS_summary/8986589

Human research participants

Policy information about studies involving human research participants and Sex and Gender in Research.

Reporting on sex and gender	Analysis using Swedish twins was stratified by sex and no differences were found. (Results are shown in Table S3). For results using published GWAS summary statistics, sex was not available so we could not stratify by sex.
Population characteristics	Quantitative twin analyses were adjusted for the covariates of sex and birth year. Covariates not available and not relevant for analyses using GWAS summary statistics.
Recruitment	For the section using the Swedish Twin Register, all twins born in Sweden between 1911 and 1985 were invited to participate. Sub-cohorts included: the Study of Twin Adults Genes and Environment (STAGE)- born 1959-1985, response rate 59.6%, n= 25387; TwinGene- born 1911-1958, response rate 46%, n=14590; and Screening Across the Lifespan of Twins – Young (SALTY)- born 1939-1958, response rate 65%, n=6605.
Ethics oversight	Ethical approval was provided by the Swedish Ethical Review Authority in Stockholm, Sweden (approval numbers: 03/224 for STAGE,2008/1735-31/3 for SALTY and 2011/463-32 for TwinGene).

Note that full information on the approval of the study protocol must also be provided in the manuscript.

Field-specific reporting

Please select the one below that is the best fit for your research. If you are not sure, read the appropriate sections before making your selection.

☒ Life sciences ☐ Behavioural & social sciences ☐ Ecological, evolutionary & environmental sciences

For a reference copy of the document with all sections, see nature.com/documents/nr-reporting-summary-flat.pdf

Life sciences study design

All studies must disclose on these points even when the disclosure is negative.

Sample size	There was no sample size calculation used. This is because we were using a large cohort of Swedish Twins (n= 28 394) and conditions that are common - asthma (8%0, allergic rhinitis (11%), eczema (7%) and gastro esophageal reflux (12%). Therefore power was not an issue for this study.
Data exclusions	The only exclusions were if one of the twins in the pair was missing becuase we could not do twin analysis, and if they were missing data on asthma, eczema, allergic rhinitis or gastro-esophageal reflux as these were the diseases under analysis.
Replication	As we are using exisiting cohort data in large datasets analyses were able to be replicated.
Randomization	This was not a randomized controlled trial but rather, a cohort using twins. Allocation was based on presence of disease or not as reported in questionnaires or recorded in administrative health data for asthma, allergic rhinitis, eczema or gastro-esophageal disease.
Blinding	Not relevant. We were using big data, hence although an analyst could look at the data to see the disease status of the participants it has no relevance as there is so much data (28 000 participants) requiring a complicated twin analysis that looking at the data would not really give an impression of what the results would procur, let alone bias it.

Reporting for specific materials, systems and methods

We require information from authors about some types of materials, experimental systems and methods used in many studies. Here, indicate whether each material, system or method listed is relevant to your study. If you are not sure if a list item applies to your research, read the appropriate section before selecting a response.

Materials & experimental systems

n/a	Involved in the study
☒	☐ Antibodies
☒	☐ Eukaryotic cell lines
☒	☐ Palaeontology and archaeology
☒	☐ Animals and other organisms
☒	☐ Clinical data
☒	☐ Dual use research of concern

Methods

n/a	Involved in the study
☒	☐ ChIP-seq
☒	☐ Flow cytometry
☒	☐ MRI-based neuroimaging
